# Pharmacogenetics of Praziquantel Metabolism: Evaluating the Cytochrome P450 Genes of Zimbabwean Patients During a Schistosomiasis Treatment

**DOI:** 10.3389/fgene.2022.914372

**Published:** 2022-06-08

**Authors:** Grace Zdesenko, Takafira Mduluza, Francisca Mutapi

**Affiliations:** ^1^ Ashworth Laboratories, Institute of Immunology and Infection Research, University of Edinburgh, Edinburgh, United Kingdom; ^2^ Ashworth Laboratories, NIHR Global Health Research Unit Tackling Infections to Benefit Africa (TIBA), University of Edinburgh, Edinburgh, United Kingdom; ^3^ Department of Biochemistry, University of Zimbabwe, Harare, Zimbabwe

**Keywords:** praziquantel, PZQ, schistosomiasis, drug metabolism, pharmacogenetics, cytochrome P450, single nucleotide polymorphisms, SNP

## Abstract

Schistosomiasis is a parasitic disease infecting over 236 million people annually, with the majority affected residing on the African continent. Control of this disease is reliant on the drug praziquantel (PZQ), with treatment success dependent on an individual reaching PZQ concentrations lethal to schistosomes. Despite the complete reliance on PZQ to treat schistosomiasis in Africa, the characterization of the pharmacogenetics associated with PZQ metabolism in African populations has been sparse. We aimed to characterize genetic variation in the drug-metabolising cytochrome P450 enzymes (CYPs) and determine the association between each variant and the efficacy of PZQ treatment in Zimbabwean patients exposed to *Schistosoma haematobium* infection. Genomic DNA from blood samples of 114 case-control Zimbabweans infected with schistosomes were sequenced using the *CYP1A2*, *CYP2C9*, *CYP2C19*, *CYP2D6*, *CYP3A4*, and *CYP3A5* genes as targets. Bioinformatic tools were used to identify and predict functional effects of detected single nucleotide polymorphisms (SNPs). A random forest (RF) model was then used to assess SNPs most predictive of PZQ efficacy, with a misclassification rate of 29%. SNPs were detected across all six genes, with 70 SNPs identified and multiple functional changes to the CYP enzymes predicted. Only four SNPs were significantly associated with PZQ efficacy using χ^2^ tests, with rs951840747 (OR: 3.61, *p* = 0.01) in the *CYP1A2* gene having the highest odds of an individual possessing this SNP clearing infection, and rs6976017 (OR: 2.19, *p* = 0.045) of *CYP3A5* determined to be the most predictive of PZQ efficacy *via* the RF. Only the rs28371702 (CC) genotype (OR: 2.36, *p* = 0.024) of *CYP2D6* was significantly associated with an unsuccessful PZQ treatment. This study adds to the genomic characterization of the diverse populations in Africa and identifies variants relevant to other pharmacogenetic studies crucial for the development and usage of drugs in these populations.

## 1 Introduction

Schistosomiasis is an ever prominent public health problem in Africa (Ross et al., 2002), with the majority of individuals affected on the continent suffering from *S*. *haematobium* and *S. mansoni* infections (Utzinger et al., 2009). Mass drug administration for the control of schistosome infections utilizes praziquantel (PZQ) as the drug of choice to reduce morbidity (Daumerie and Savioli, 2013). PZQ is racemic, with the (R)-PZQ enantiomer possessing antischistosomal activity and (S)-PZQ contributing to PZQ’s known side effects (Meyer et al., 2009). The efficacy of PZQ treatment is determined by two outcomes: egg reduction rate (ERR), determined by the reduction in mean number of eggs excreted in urine or stool (depending on the schistosome species) from pre-PZQ to post-PZQ treatment, and the cure rate (CR) which gives the proportion of egg-positive individuals pre-PZQ treatment who become negative for schistosomiasis post-PZQ treatment (Zwang and Olliaro, 2014). Our previous study showed that various factors, including PZQ metabolism, contribute towards variable CRs (Zdesenko and Mutapi, 2020). Like several African countries, Zimbabwe has been administering PZQ as part of a national helminth control program for over 10 years. We have previously reported that the national program has significantly reduced infection prevalence (Mduluza et al., 2020), however there have been multiple reports of hotspots of schistosomiasis infection across other areas of Africa (Kittur et al., 2017; Kittur et al., 2019). Consequently, it is critical to understand the reasons for the persistence of infection, especially if the implementation of this knowledge can aid in improving control of this disease. In this study, we highlight the paucity of pharmacogenetic studies in African populations, focusing on Zimbabwe, where PZQ is heavily used. Such studies are important to inform on drug failures (Drew, 2016) and contribute towards the global efforts to eliminate schistosomiasis, a goal recently highlighted in the new WHO NTD roadmap (WHO, 2020).

Drug metabolism is mediated by the cytochrome P450 (CYP) enzymes, and genetic polymorphisms in CYPs have already been linked to inter-individual variation in drug metabolism in numerous drug efficacy and toxicity studies (Evans and Relling, 1999). Due to random mutation, meiotic recombination, and genetic drift, the African continent has greater genetic diversity than any other continental population (Tishkoff et al., 2009; Rajman et al., 2017). Nonetheless, African populations are the least studied in terms of pharmacogenetics for the majority of drug treatments (Radouani et al., 2020). Therefore, the lack of characterization of the pharmacogenetics associated with PZQ metabolism needs to be addressed (Mduluza and Mutapi, 2017). The distribution of CYP variants differs substantially between populations, thus pharmacogenetic studies conducted in European populations are not always representative of other ethnicities, including the more genetically diverse African populations (Masimirembwa and Hasler, 1997). Therefore, by characterising single nucleotide polymorphisms (SNPs) in the CYP enzymes that are key in mediating PZQ metabolism we have the potential to inform on associations of SNPs with an individual’s treatment response (Evans and Johnson, 2001; McLeod and Evans, 2001). SNPs that potentially decrease or inactivate the CYP enzyme may reduce the metabolism of active PZQ to its inactive metabolites, sustaining a lethal PZQ concentration to the schistosomes, and increasing the likelihood of clearing infection (Zimmermann et al., 2007; Nleya et al., 2019). Conversely, an increased rate of PZQ metabolism may result in systemic PZQ concentrations that do not reach or exceed the lethal levels required to clear schistosome infection.

To date, six CYPs*: CYP1A2*, *CYP2C9*, *CYP2C19*, *CYP2D6*, *CYP3A4*, and *CYP3A5* have been identified to be involved in the PZQ metabolism pathways (Bonate et al., 2018; Kapungu et al., 2020), as well as being responsible for metabolising 90% of market drugs (Slaughter and Edwards, 1995; Wilkinson, 2005). Each CYP has varying contributions to PZQ metabolism, and the conclusive pathway does not always include CYP2D6. However, as the impact of CYP2D6 in African populations is scarce we included this enzyme to provide valuable information for, not just this study, but other pharmacogenetic analyses. Thus, in this study we focused on the genetic variants of these CYPs and their association with PZQ clearance of *S*. *haematobium* infections in Zimbabweans resident in a schistosome endemic area. We aimed to characterize the SNPs present in these six CYPs, their frequency in the Zimbabwean population, and be the first to assess their association with PZQ efficacy in this genetically diverse population.

## 2 Materials and Methods

### 2.1 Study Design, Demographic Characteristics, and Measures of Praziquantel Efficacy

The case-control study aimed to investigate whether 1) there was genetic variation in the CYP450 enzymes involved in PZQ metabolism, 2) determine if there was an association between each variant and the efficacy of PZQ treatment in a Zimbabwean population exposed to *S. haematobium* infection, and 3) predict an individual’s PZQ treatment outcome based on all the SNPs detected. The samples used in this study were part of a larger study on the immunological effects of schistosomiasis conducted in the Mashonaland East province, which has a *S. haematobium* prevalence of 30.4% (Mduluza et al., 2020). Samples included in the current study were from two districts in this province, Mutoko and Murewa, and details of their parasitology and blood sampling have been previously published (Osakunor et al., 2020). This case-control study was designed in the context of a pharmacogenetic evaluation, in which the subjects are divided into those with a positive response to PZQ who cleared schistosome infection, and those with negative or no response who did not clear schistosome infection. These groups then constitute as cases and controls that could be related to the treatment phenotype (Weiss et al., 2001). As this is a novel and exploratory study there were no baseline studies to inform sample size calculations, but samples were selected to ensure matching on treatment outcome, sex, and age. This matching was essential in the study to reduce the impact of factors known to affect the efficacy of treatment (e.g., host pre-treatment infection intensity, all individuals fed prior to treatment), minimizing all known heterogeneities that would affect the drug efficacy. Furthermore, as confirmed by the initial health assessment, none of the participants were on concomitant drug treatments to remove the risk of drug-drug interactions.

The characteristics and matching of the 114 participants selected for this study are described in Supplementary Table S1. All participants were positive for *S. haematobium* infection and were treated at baseline with PZQ. These individuals were then followed up 6 weeks later to obtain the post-treatment efficacy outcome. The efficacy of PZQ treatment was determined by the ERR, determined by the reduction in mean number of eggs excreted in urine from pre-PZQ to post-PZQ treatment (Zwang and Olliaro, 2014). At the 6-weeks efficacy check, the subjects were divided into either: 1) negative and cleared schistosome infection (*n* = 57, 100% ERR), indicative of a successful treatment, or 2) still positive for schistosomes and did not clear infection (*n* = 57, <100% ERR), indicative of an unsuccessful treatment; controlling each sample for age, sex, and initial egg burden. To ensure treatment compliance each individual was checked by a health worker to confirm they had swallowed the tablet.

### 2.2 DNA Extraction and Target Sequencing

Genomic DNA was extracted from the blood samples using QIAamp DNA MicroKit (Qiagen, GmbH, Germany), according to manufacturer’s protocol. The DNA samples were shipped on dry ice for library preparation and targeted metagenomic sequencing to BGI (Beijing Genomics Institute, Shenzhen, China). Briefly, DNA samples were quantified using the Qubit fluorometer (ThermoFisher Scientific, New Territories, Hong Kong) and the NanoDrop™ spectrophotometer (ThermoFisher Scientific, New Territories, Hong Kong). The integrity and purity of DNA was assessed by a 1% agarose gel electrophoresis. Qualified DNA was randomly fragmented by Covaris technology (150 bp–250 bp fragments) (Covaris, Woburn, United States) and end repair of DNA fragments was performed with an “A” base added to 3′-end of each strand. Adapters were then ligated to both ends of the end repaired/dA tailed DNA fragments. A hybridisation-based target enrichment and selective amplification of the size-selected DNA fragments was performed using ligation-mediated PCR for the target CYP gene regions. PCR products were purified with the AxyPrep Mag PCR clean up Kit (Axygen Scientific, Taipei, Taiwan). Adapter-ligated DNA fragments were separated by electrophoresis through a 2% agarose gel to recover the target fragments, purified using the QIAquick Gel Extraction kit (Qiagen, GmbH, Germany), and circularised to produce DNA nanoballs. Targeted enrichment of the *CYP1A2*, *CYP2C9*, *CYP2C19*, *CYP2D6*, *CYP3A4*, and *CYP3A5* genes was performed using a custom Agilent SureSelect Target Enrichment Kit (Agilent Technologies, Santa Clara, United States) according to the manufacturer’s protocol. Sequencing probes for the six targeted genes were custom designed using Agilent’s SureDesign tool (www.agilent.com/genomics/suredesign) to include all exons, introns, intergenic, and promoter regions according to the tools reference databases. After initial amplification of the target regions, different pairs of index primers were added to each sample in a second thermocycling step with barcode recognition. This produced a library of DNA amplicons representing individuals for sequencing and allowed multiple libraries to be pooled together to be sequenced in the same run. Each captured library was loaded on the Illumina Hiseq 4000 platform (Illumina, San Diego, United States) and the raw image files were processed by DNBseq base calling software with default parameters. The sequence data of each individual was generated as paired-end reads and stored in FASTQ format.

### 2.3 Bioinformatic Analysis

Variant Call Format (VCF) file from the raw sequence data was produced according to Supplementary Procedure S1, with alignment ensuring SNP regions of interest were covered [as selected from a pharmacogenetic review of African populations (Zhou et al., 2017)], in addition to the full genes including exon/intron boundaries and flanking regions. Each SNP was determined to have an average coverage of >87X per sample. Each SNP was assigned an ID and designated as either: 1) reported CYP allele, 2) reported rs-code, or 3) a novel SNP. The SNPs were designated a novel ID if no corresponding SNP position was found in dbSNP (Sherry et al., 2001) or Ensembl (Hubbard et al., 2007), according to the human genome assembly GRCh38.

### 2.4 Variant Analysis and Transcriptional Prediction

The SNPs were assigned an official allele nomenclature using The Human Cytochrome P450 Allele Nomenclature Database (PharmVar) (Gaedigk et al., 2018) and SNPedia (Cariaso and Lennon, 2012), with the remaining variants designated an rs-number using dbSNP (Sherry et al., 2001) and Gnomad (https://gnomad.broadinstitute.org/). The nucleotide changes and the amino acid change of each SNP was obtained using Mutalyzer (Wildeman et al., 2008) and Ensembl Variant Effect Predictor (Hubbard et al., 2007), respectively. A predicted effect on protein function of missense variants was obtained using the SIFT tool (Ng and Henikoff, 2003). SIFT predictions are only shown for complete Ensembl proteins. For non-coding variations, the Genome-Wide Annotation of Variants (GWAVA) (Ritchie et al., 2014) scoring tool was used to predict the functional impact of the SNP. SNPs with a MAF <5% were removed from subsequent analysis but were reported in Supplementary Table S2 due to lack of power to detect an association in this sample size.

### 2.5 Statistical Analysis

Univariate association of SNP to PZQ efficacy was performed using PLINK (Purcell et al., 2007) to visualize and interpret results. Each SNP’s minor allele *a* and major allele *A* was represented as a contingency table of PZQ treatment outcome by either genotype count (*aa*, *Aa*, and *AA*) or allele count (*a* and *A*) according to genetic models described by Clarke et al. (2011). PLINK assessed these genetic models using χ^2^ tests, with the χ^2^_Yates_ calculated to assess possible type I error for further discussion. χ^2^ tests were used to assess significant differences between the MAF of the SNPs against three populations; the African and European populations as reported by 1,000 Genomes Project (Auton et al., 2015), and the Zimbabwean population based on a limited number of studies conducted (Matimba et al., 2009; Dandara et al., 2004; Dandara et al., 2001; Matimba et al., 2008; Masimirembwa et al., 1995; Roy et al., 2005) using IBM SPSS Statistics v.25 (https://www.ibm.com/uk-en/analytics/spss-statistics-software). The 1,000 Genomes Project was selected as a comparison as it contained the most representative database of eastern African populations and was therefore the most relevant to this study. Quantile plots were used to discern whether the *p*-values followed the null distribution, with the observed −log_10_ (*p*-values) of each SNP plotted against expected −log_10_ (*p*-values) (Supplementary Figure S1). The ORs were presented with a 95% CI and all statistical significance was set at *p* ≤ 0.05. LAMPLINK was used to detect statistically significant combinatorial interactions between ≥2 SNPs using the procedure set out by Terada et al. (2016). Linkage disequilibrium (LD) and Hardy–Weinberg equilibrium (HWE) between pairs of SNPs on the same chromosome were assessed using the Haploview software (http://broad.mit.edu/mpg/haploview, Version 4.1) (Barrett et al., 2005). As deviations from HWE may be indicative of genotyping or genotype-calling errors, SNPs with HWE (*P* < 1 × 10^−4^) were excluded from analysis (Marees et al., 2018). The LD test statistics and the strength of LD was designated according to Supplementary Table S3. To assess non-random associations between SNPs in LD, the presence of haplotypes was evaluated in Haploview, with blocks indicating where there was statistical evidence of strong co-inheritance. Haplotype blocks were defined based on the Gabriel method (upper 95% CI of the D′ value is ≥0.98, and the lower 95% CI is ≥0.7; MAF, >5%) (Gabriel et al., 2002). The random forest (RF) model was built a series of classification trees by randomly sampling a subset of the subjects to form an out-of-bag (OOB) data set. The RF then randomly selects and searches, building a tree for the best SNP predictive of a PZQ treatment outcome. Further information regarding the development of the model is described in Supplementary Figure S2.

## 3 Results

### 3.1 Detected Single Nucleotide Polymorphisms

#### 3.1.1 Minor Allele Frequency

A total of 70 Single Nucleotide Polymorphisms (SNPs) with an allele frequency in the population >5% were identified, with the Minor Allele Frequency (MAF) and variant characteristics of each SNP displayed in Table 1, including the predicted or reported functional change of each SNP to its respective CYP450 enzyme. The allele frequencies of each SNP (Table 1; Column EXP) were in HWE, with 10 common CYP alleles, 34 rs-number SNPs, and 26 novel SNPs to be evaluated. Comparisons between the MAF of the different populations showed that 17.1% of detected SNPs were found at a significantly higher frequency in this Zimbabwean study population than expected in an African population. These significantly different SNPs were not detected in the African populations at all (MAF = 0%), yet this study detected those same SNPs with a frequency as high as 99.1% in comparison, exemplifying the scarcity of relevant frequency data in Zimbabwe. Furthermore, although the SNPs detected for genetic evaluation are described as the minor allele, 4.3% of variants in this study had a MAF greater than 90%. This included rs2040992 of *CYP3A5*, which was detected as a completely homozygous SNP in this population but was not significantly different from the frequency reported in African populations. Yet, 4.3% of detected SNPs were also found to be significantly different to other genetic studies conducted in Zimbabwe, with 2.8% of detected SNPs significantly lower in the study population compared to the reported frequency in Zimbabwe. This included the CYP3A5*3 polymorphism in which this study detected a significantly lower frequency than in European populations and other Zimbabwean studies reported, with a −78.5% and −61.8% decrease, respectively. The significantly lower MAF detected in this study compared to other Zimbabwean study populations only occurred for one additional SNP: rs4917623 of *CYP2C19*. For the remaining common CYP alleles, there were no significant differences in MAF detected in this study between either the African or Zimbabwean populations.

**TABLE 1:** Characteristics and minor allele frequency (MAF) of the 70 single nucleotide polymorphisms (SNPs) detected in the Zimbabwean subjects. Each SNP had a MAF > 5%, and the predicted and reported functional changes of each cytochrome P450 (CYP) were described.

DNA strand	Enzyme	Identifier	Allele	MAF (%)				Chromosome position	Nucleotide change	Amino acid change	SIFT/GWAVA prediction	Reported enzyme activity	Variant consequence	Region
				EXP	AFR	ZIM	EUR							
1	*CYP1A2*	rs2069514	CYP1A2*1C	29.39	31.32	—	1.99*	74745879	G > A	—	-/DEL	Decreased (Nakajima et al., 1999)	Upstream Gene	—
		rs762551	CYP1A2*1F	53.51	56.2	57	67.99**	74749576	C > A	—	-/DEL	Increased (Lu et al., 2020)	Intron	I1
		rs2069526	CYP1A2*1K	11.84	12.33	—	2.39*	74749000	T > G	—	-/TOL	Decreased (Al-Ahmad et al., 2017)	Intron	I1
		NOVEL_74753482	Novel-1	12.72	—	—	—	74753482	G > A	—	-/TOL	—	Intron	I6
		NOVEL_74753485	Novel-2	86.84	—	—	—	74753485	G > C	—	-/TOL	—	Intron	I6
		NOVEL_74753512	Novel-3	13.16	—	—	—	74753512	C > T	—	-/TOL	—	Intron	I6
		NOVEL_74753515	Novel-4	7.02	—	—	—	74753515	G > A	—	-/TOL	—	Intron	I6
		NOVEL_74747828	Novel-5	8.33	—	—	—	74747828	C > T	—	-/DEL	—	Upstream Gene	-
		NOVEL_74747713	Novel-6	6.14	—	—	—	74747713	A > G	—	-/DEL	—	Upstream Gene	-
		NOVEL_74747716	Novel-7	7.46	—	—	—	74747716	C > T	—	-/DEL	—	Upstream Gene	-
		NOVEL_74747757	Novel-8	10.09	—	—	—	74747757	G > A	—	-/DEL	—	Upstream Gene	-
		rs1022705765	rs1022705765	10.53	0*	—	—	74753493	G > A	—	-/TOL	—	Intron	I6
		rs1450415112	rs1450415112	9.65	0*	—	—	74753511	A > G	—	-/TOL	—	Intron	I6
		rs951840747	rs951840747	10.09	0*	—	—	74753480	C > T	—	-/TOL	—	Intron	I6
	*CYP2C9*	rs2256871	CYP2C9*9	14.04	8.17	13	0.1*	94949217	A > G	His251Arg	DEL/DEL	Normal (Matimba et al., 2009)	Missense	E5
		NOVEL_94956743	Novel-9	7.46	-	—	—	94956743	T > C	—	-/TOL	—	Intron	I5
		NOVEL_94977838	Novel-10	6.14	-	—	—	94977838	C > T	—	-/TOL	—	Intron	I6
		rs2017319	rs2017319	13.60	12.03	10	0.2*	94988878	C > T	Ala441 (3D)	-/TOL	—	Synonymous	E9
		rs75541073	rs75541073	16.23	19.14	—	0.1*	94948233	G > A	—	-/TOL	—	Intron	I4
		rs9332127	rs9332127	14.47	15.81	—	0.1*	94947714	G > C	—	-/TOL	Decreased (Wang et al., 2008; Gu et al., 2010)	Intron	I3
		rs9332232	rs9332232	13.60	11.95	11	0.2*	94986275	T > C	—	-/TOL	—	Intron	I8
		rs9332241	rs9332241	7.02	3.78	5	0*	94989116	C > T	—	-/TOL	—	3′ UTR	E9
	*CYP2C19*	rs4244285	CYP2C19*2	16.23	17.02	15	14.51	94781859	G > A	Pro218	-/TOL	Decreased (Dandara et al., 2001)	Synonymous	E5
		rs12248560	CYP2C19*17	15.79	23.52	—	22.37	94761900	C > T	—	-/DEL	Increased (Sim et al., 2006; Gawrońska-Szklarz et al., 2012)	Upstream Gene	-
		rs7902257	CYP2C19*27	13.60	8.25	—	0.1*	94761665	G > A	—	-/-	Uncertain (Helsby and Burns, 2012)	Upstream Gene	-
		NOVEL_94779010	Novel-11	6.58	—	—	—	94779010	G > A	—	-/TOL	—	Intron	I3
		rs17879992	rs17879992	11.84	9.76	—	6.96	94775871	T > C	—	-/TOL	—	Intron	I3
		rs17884938	rs17884938	9.21	5.82	—	0.2*	94780934	T > A	—	-/TOL	—	Intron	I4
		rs17885567	rs17885567	5.70	5.60	10	0*	94850227	C > T	—	-/TOL	—	Intron	I8
		rs4917623	rs4917623	10.96	19.82	22**	50.8**	94849811	T > C	—	-/TOL	—	Intron	I7
		rs4986894	rs4986894	14.91	16.26	15	14.51	94762608	T > C	—	-/DEL	—	Upstream Gene	-
		rs76267522	rs76267522	5.26	2.34	—	0.1*	94780959	T > C	—	-/TOL	—	Intron	I4
−1	*CYP2D6*	rs28371706	CYP2D6*17	16.67	21.80	20	0.2*	42129770	G > A	Thr107Ile	TOL/-	Decreased (Masimirembwa et al., 1996)	Missense	E2
		rs17002853	rs17002853	5.70	0*	—	0.1*	42128325	A > G	Leu231Pro	DEL/-	—	Missense	E5
		rs28371702	rs28371702	75.88	77.08	29*	54.77*	42129950	A > C	—	-/-	Normal/Decreased (Zhang et al., 2021)	Intron	I1
	*CYP3A4*	NOVEL_99761942	Novel-12	7.89	—	—	—	99761942	T > C	—	-/TOL	—	Intron	I11
		NOVEL_99764101	Novel-13	7.89	—	—	—	99764101	T > C	—	-/TOL	—	Intron	I9
		NOVEL_99766252	Novel-14	5.26	—	—	—	99766252	C > T	—	-/TOL	—	Intron	I10
		NOVEL_99764034	Novel-15	16.23	—	—	—	99764034	C > T	—	-/TOL	—	Intron	I11
		NOVEL_99764099	Novel-16	8.33	—	—	—	99764099	C > T	—	-/TOL	—	Intron	I12
		NOVEL_99762108	Novel-17	16.23	—	—	—	99762108	T > C	Ile396Val	TOL/DEL	—	Missense	E11
		NOVEL_99762115	Novel-18	7.46	—	—	—	99762115	C > T	Val393	-/TOL	—	Synonymous	E11
		NOVEL_99762116	Novel-19	7.46	—	—	—	99762116	A > G	Val393Ala	TOL/DEL	—	Missense	E11
		NOVEL_99784449	Novel-20	15.35	—	—	—	99784449	A > G	—	-/DEL	—	Upstream Gene	—
		NOVEL_99784471	Novel-21	11.40	—	—	—	99784471	T > A	—	-/DEL	—	Upstream Gene	—
		rs1006181087	rs1006181087	8.33	0*	—	—	99762118	C > T	Val392	-/TOL	—	Synonymous	E11
		rs12721622	rs12721622	9.65	4.39	—	0*	99768236	A > T	—	-/TOL	—	Intron	I7
		rs1479820461	rs1479820461	99.12	0*	—	0*	99783699	G > C	—	-/DEL	—	ntron	I1
		rs2687110	rs2687110	62.28	59.23	—	99.7**	99773128	A > T	—	-/TOL	-	Intron	I3
		rs2687116	rs2687116	23.25	27.91	—	97.02**	99768320	C > A	—	-/TOL	Decreased (Panczyk, 2014)	Intron	I7
		rs28988583	rs28988583	11.84	8.70	—	2.09*	99769086	A > G	—	-/TOL	—	Intron	I6
		rs3735451	rs3735451	82.02	81.39	—	9.94*	99758352	T > C	—	-/TOL	—	Intron	I13
		rs746971934	rs746971934	8.33	0*	—	—	99762120	C > A	Val392Leu	DEL/DEL	—	Missense	E11
		rs778270963	rs778270963	15.79	0*	—	—	99762101	C > A	Ser398Ile	TOL/-	—	Missense	E11
		rs915268104	rs915268104	6.58	0*	—	—	99783718	C > T	—	-/DEL	—	Intron	I1
	*CYP3A5*	rs776746	CYP3A5*3	15.79	18	77.6**	94.33**	99672916	T > C	—	-/-	Inactive (Kuehl et al., 2001; Sanghavi et al., 2017)	Intron	I3
		rs10264272	CYP3A5*6	15.35	15.43	22	0.3*	99665212	C > T	Lys208	-/DEL	Inactive (Kuehl et al., 2001; Sanghavi et al., 2017)	Synonymous	E7
		NOVEL_99672254	Novel-22	15.79	—	—	—	99672254	G > A	—	-/TOL	—	Intron	I4
		NOVEL_99672211	Novel-23	97.81	—	—	—	99672211	G > C	—	-/TOL	—	Intron	I4
		NOVEL_99672251	Novel-24	5.26	—	—	—	99672251	C > T	—	-/TOL	—	Intron	I4
		NOVEL_99672285	Novel-25	15.35	—	—	—	99672285	C > T	—	-/TOL	—	Intron	I4
		NOVEL_99666556	Novel-26	6.14	—	—	—	99666556	A > G	—	-/DEL	—	Intron	I6
		rs1039108105	rs1039108105	9.21	0*	—	0*	99656082	G > C	—	-/TOL	—	Intron	I10
		rs1458424958	rs1458424958	5.26	0*	—	0*	99673203	A > G	—	-/TOL	—	Intron	I3
		rs1462057054	rs1462057054	16.67	0*	—	0*	99672249	A > G	—	-/TOL	—	Intron	I4
		rs2040992	rs2040992	100	99.92	—	98.61	99664949	G > A	—	-/DEL	—	Intron	I7
		rs41303322	rs41303322	10.53	11.88	—	0*	99664999	T > C	—	-/TOL	—	Intron	I7
		rs4646453	rs4646453	9.21	8.77	—	1.89*	99662739	C > A	—	-/TOL	—	Intron	I13
		rs6976017	rs6976017	20.18	13.99	—	3.08*	99652376	G > A	—	-/TOL	—	Intron	I11
		rs8175345	rs8175345	9.65	8.09	—	0*	99672695	G > A	—	-/DEL	—	Intron	I3

* and ** represents a significantly higher or lower difference in the minor allele frequency (MAF) in this Zimbabwean (ZIM) study population compared to the reported MAF in the African (AFR)/European (EUR) population in the 1,000 Genomes Project using Pearson’s chi-square tests.

SIFT, sorting Intolerant From Tolerant; GWAVA, Genome-Wide Annotation of Variants; DEL, Deleterious effect on protein function; TOL, Tolerated effect on protein function. Region described by: Exon (E) and Intron (I). CYP, Cytochrome P450 enzyme.

Interestingly, for the allele frequencies detected in the Zimbabweans in this study, only 52.9% had been previously detected in a European population. In fact, 87% of comparable alleles in this Zimbabwean population had significantly different frequencies than in their corresponding European populations. Regarding comparisons of this study population to European populations, of the 10 common CYP alleles identified in this study, 60% were found at a significantly higher percentage in the Zimbabwean study population than expected based on European populations. These CYP alleles included the CYP1A2*1C and CYP1A2*1K variants which have been reported to decrease CYP function yet were found at a +27.4% and +9.45% high frequency than observed in Europeans. If present in the Zimbabwean population at a significantly higher frequency than what is presented by European studies, these two variants could decrease CYP metabolism which could lead to higher exposure of an administered drug in Zimbabweans and impact the success and safety of a drug treatment.

#### 3.1.2 Predicted Functional Consequences

Alterations to the nucleotide sequence by SNPs can impact the resultant protein translation. These changes can affect protein function and therefore impact a drug’s metabolism, including the compromise and enhancement of protein function, or having no effect at all. In this study, there were 7 missense, 5 synonymous, 47 intronic, one 3′ UTR, and 10 upstream gene variants detected, with each classification impacting the consequent DNA sequence differently. In this study, 3 missense variants were predicted by SIFT to be a deleterious change, and therefore possibly damaging to the function of their respective CYP enzyme. These potentially deleterious variants included CYP2C9*9 (His251Arg) of the *CYP2C9* gene, rs17002853 (Leu231Pro) in *CYP2D6* gene, and rs746971934 (Val392Leu) in the *CYP3A4* gene. These SNPs alterations damaged the translated protein, which could eventually affect each enzyme’s metabolising capacity. Conversely, a further 4 missense variants were predicted to result in a tolerated amino acid change, with no alteration to protein function and overall capacity of the enzyme. These tolerated changes included the CYP2D6*17 polymorphism, in which SIFT predicted Thr107Ile to have no impact *CYP2D6* function, as well as Novel-17 (Ile396Val), Novel-19 (Val393Ala), and rs778270963 (Ser398Ile) of the *CYP3A4* enzyme. However, as the SIFT predictor tool is not always definitive of resultant protein function, the GWAVA prediction was also reported. For the novel missense findings described above, GWAVA predicted they may be deleterious to *CYP3A4* function, rather than tolerated as SIFT described.

For the synonymous SNPs detected that do not change the encoding amino-acid, two SNPs were already well reported to impact CYP function; the CYP2C19*2 and CYP3A5*6 alleles. These SNPs are reported to decrease or inactivate their respective CYPs function, however only the CYP3A5*6 was predicted to be deleterious by GWAVA. The remaining 3 synonymous SNPs were predicted to be tolerated, with no protein translation to alter the function of the CYPs, including one novel SNP: Novel-19 (Val393) of *CYP3A4*. From the remaining intronic and upstream gene variants whose functional effect could not be predicted by SIFT, the GWAVA algorithm was used. GWAVA predicted that a further 15 SNPs possibly damaged their respective CYP function, of which three were reported CYP alleles (CYP1A2*1C, CYP1A2*1F, and CYP2C19*17) whose alterations to CYP function are already well described. A further seven possibly damaging SNPs were novel discoveries (Novel-5, Novel-6, Novel-7, Novel-8, Novel-21, Novel-20, and Novel-26) and five were reported variants (rs1479820461, rs2040992, rs4986894, rs8175345, and rs915268104), yet no published studies in relation to their impact on CYP function have been conducted.

### 3.2 Single Nucleotide Polymorphisms Associations With Praziquantel Efficacy

**FIGURE 1:**
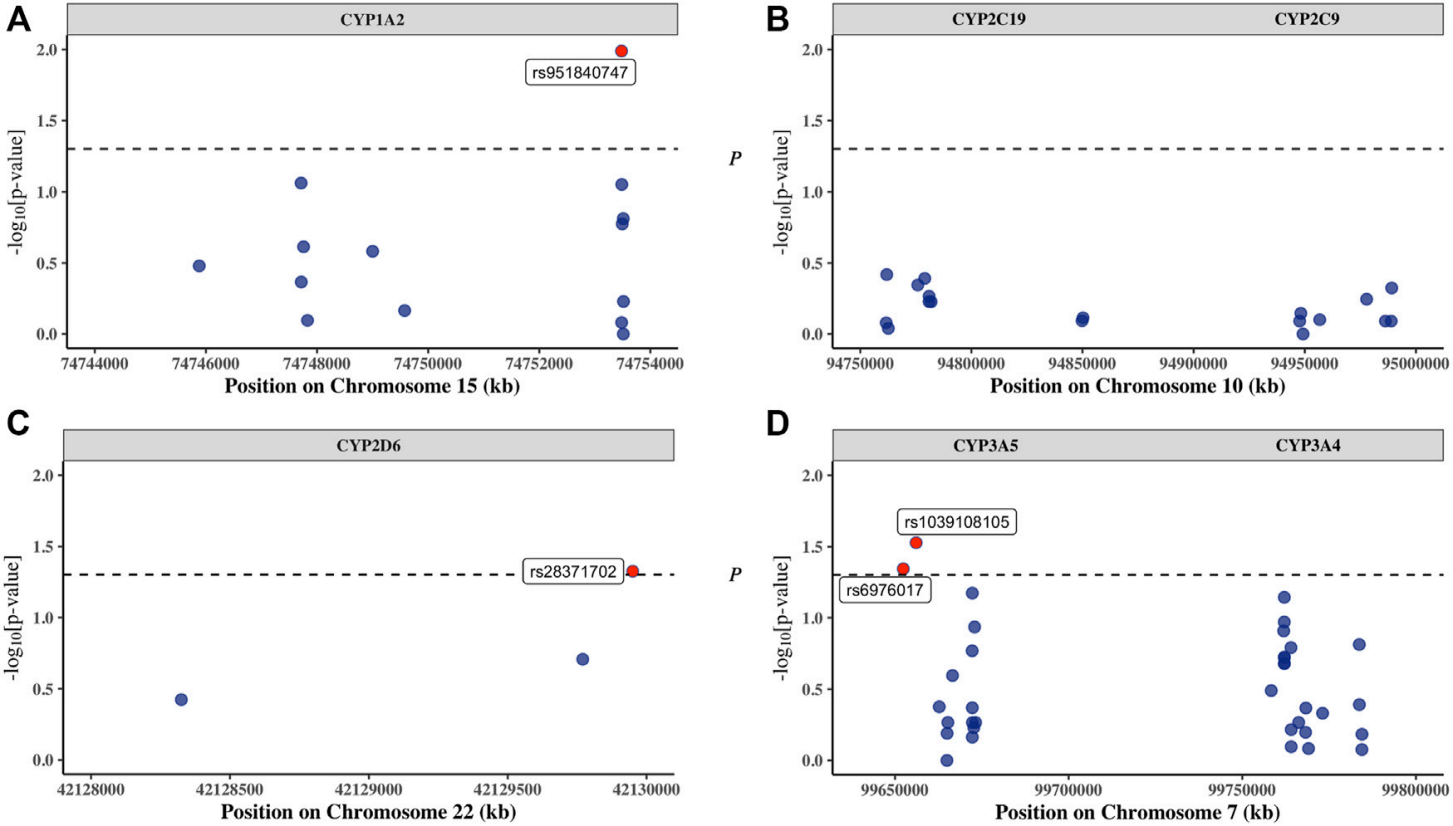
The position of each single nucleotide polymorphism (SNP) on their respective chromosome versus −log10 (*p*-value) is displayed for **(A)** *CYP1A2*, **(B)** *CYP2C9/19*, **(C)** *CYP2D6*, and **(D)** *CYP3A4/5*. The threshold for significance was set at *p* = 0.05 (black dotted line), with SNPs significantly associated with PZQ efficacy (red dots) labelled with their SNP identification.

Tests of association between SNP and PZQ efficacy was assessed using multiple genetic models. Independent association of the 70 SNPs with PZQ efficacy can be found in Supplementary Table S4, and a quantile plot of the genotypic associations was produced (Supplementary Figure S1). This indicates little evidence of an overall systematic bias yet displays a slight deviation from the null hypothesis (*y* = *x*), suggesting a small number of SNPs possessed stronger associations with PZQ efficacy than expected by chance as indicated by the slight upward curvature near the tail of the distribution. The association to PZQ efficacy was driven by SNPs in the *CYP1A2*, *CYP2D6*, and *CYP3A5* genes, with the *p*-values of SNPs above the threshold for significance labelled in Figure 1 to ascertain significant SNP deviations.

To further determine the mechanism behind the SNP deviations from the null hypothesis, hetero/homozygous genotypes were tested for an association with PZQ efficacy, with the results of each SNPs significance in Supplementary Table S5. Overall, 4 SNPs were found to have a significant association with PZQ efficacy (Table 2), with multiple genetic models conducted to best determine the genetic mechanism behind the association. To aid further discussion of the discovery of significantly associated SNPs, results of χ^2^_Yates_ test representing an adjusted *p*-value accounting for a continuity correction can be seen in Supplementary Table S6.

**TABLE 2:** The single nucleotide polymorphisms (SNPs) found to be significant associated with an individual clearing infection *via* basic association analysis. Association tests were used to determine the genotype significantly associated with PZQ efficacy using PLINK; these are listed below each genetic model. The frequency of the allele/genotype for each model is listed, with the frequency separated by treatment outcome.

Enzyme	SNP	MAF (%)		Genotypic association							Allelic association				Cochran-armitage trend				Dominant model				Recessive model			
				*P*			Genotype	*AA vs. aA vs. aa*			χ2 test		*A vs. a*		T^2^ test		*A vs. a*		χ2 test		aA + AA vs. aa		χ2 test		AA vs. aa + aA	
		*NC*	*C*	*DF*	FE	χ^2^		*P*	*NC*	*C*	*DF*	*P*	NC	C	*DF*	*P*	NC	C	DF	P	NC	C	DF	P	NC	C
*CYP1A2*	rs951840747	2.632	7.456	1	**0.018**	**0.010**	***CT***	**0.010** ^*^	0/6/51	0/17/40	1	**0.016** ^*^	6/108	17/97	1	**0.010**	6/108	17/97	1	0.010*	6/51	17/40	1	NA	0/57	0/57
							*TT*	NA																		
*CYP2D6*	rs28371702	40.351	35.326	2	**0.031**	**0.047**	***AC***	**0.014** ^*^	37/18/2	25/31/1		0.089	92/22	81/33		0.061	92/22	81/33		0.559	55/2	56/1		0.024*	37/20	25/32
							***CC***	**0.024** ^*^																		
*CYP3A5*	rs1039108105	2.632	6.579	1	0.052	**0.030**	***GC***	**0.030**	0/6/51	0/15/42		**0.039**	6/108	15/99		**0.030**	6/108	15/99		0.030	6/51	15/42		NA	0/57	0/57
							*CC*	NA																		
	rs6976017	9.211	10.965	2	**0.041**	**0.045**	***GA***	**0.050**	3/15/39	0/25/32		0.509	21/93	25/89		0.489	21/93	25/89		0.176	18/39	25/32		0.079	3/54	0/57
							*AA*	0.079																		

Bold indicates the SNP genotype that were determined to be significantly associated with PZQ efficacy. The significance threshold is a *p* ≤ 0.05, with the * indicating a *P*_*adj*_ ≤ 0.05 obtained from χ^2^_Yates_ test representing an adjusted *p*-value accounting for a continuity correction.

FE, Fishers Exact Test; CYP, Cytochrome P450 enzyme; NC, Not cleared group; C, Cleared group.

**FIGURE 2:**
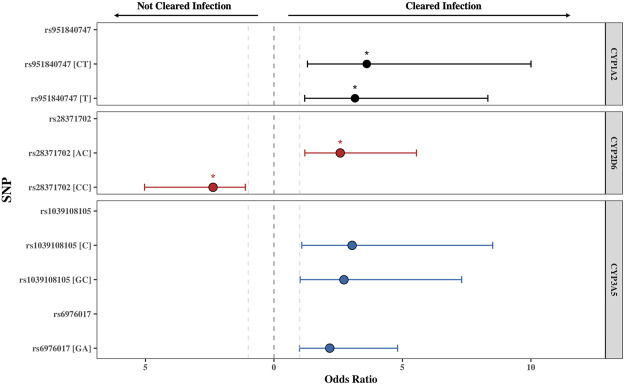
The odds ratio (OR) of the single nucleotide polymorphisms (SNPs) found to be significantly associated with PZQ efficacy for *CYP1A2, CYP2D6*, and *CYP3A5*. The threshold for significance was set at *p* ≤ 0.05, with the * indicating a *P*_*adj*_ ≤ 0.05 obtained from χ^2^_Yates_ test representing an adjusted *p*-value accounting for a continuity correction. All SNPs displayed were below this threshold. The grey lines represent OR = 1, representing no impact of the SNP on PZQ efficacy. The error bars illustrate the 95% confidence interval. The genotype or allele of each SNP is displayed in parentheses, illustrating whether the OR represents the allelic, heterozygous genotype, or homozygous genotype for the alternate allele.

Each SNPs respective odds ratio (OR) towards PZQ efficacy was also calculated. The OR of all SNPs were log-transformed to normalize their distribution and are displayed in Supplementary Figures S3, S4, with the SNPs presented to either increase odds of clearing or not clearing infection across all six genes. Of the 4 SNPs significantly associated with PZQ efficacy, the OR of an individual clearing or not clearing infection based on the presence of the SNP is visualised in Figure 2.

**FIGURE 3:**
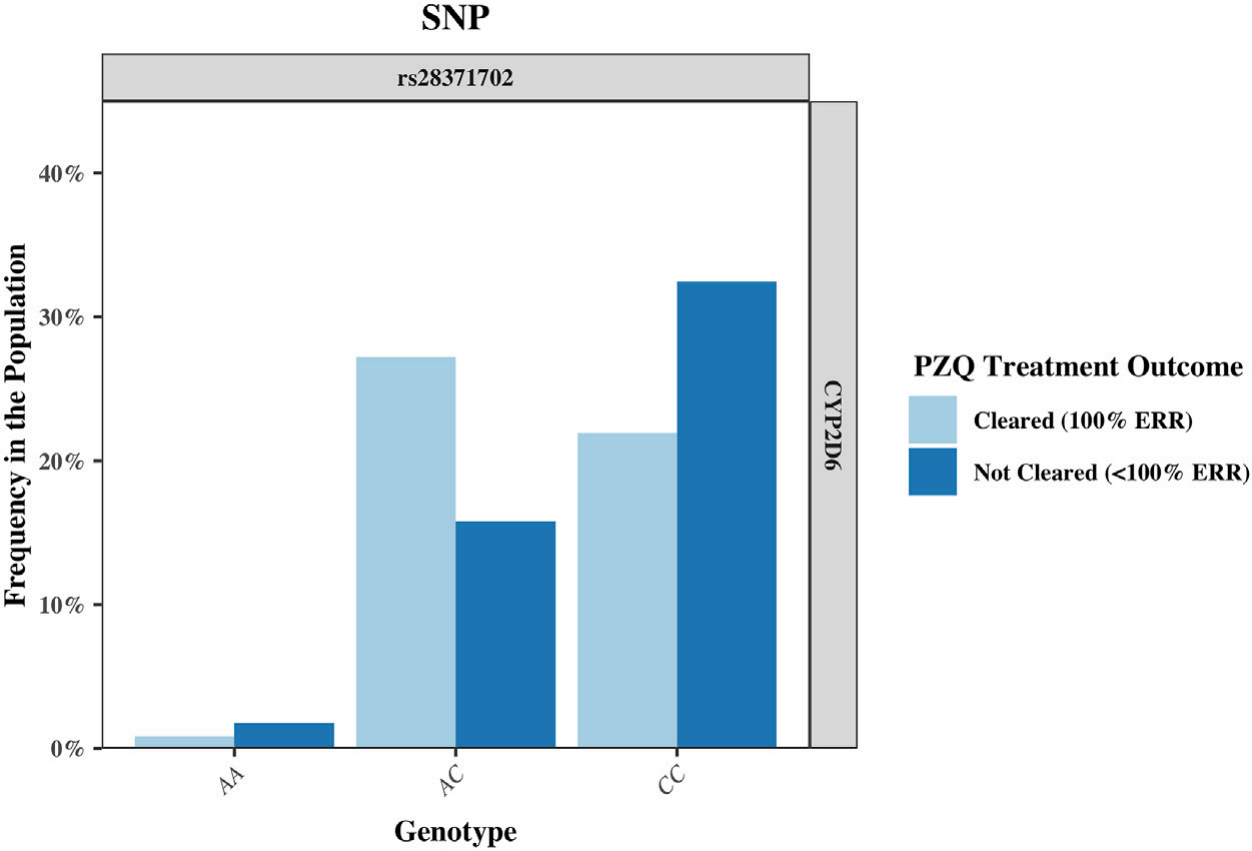
The frequency of the single nucleotide polymorphism (SNP) rs28371702 in the CYP2D6 gene, separated by genotype and sub-divided by PZQ treatment outcome. The genotype for the rs28371702 is as follows: AA (reference allele), AC (heterozygous for alternate allele), and CC (homozygous for alternate allele). PZQ: Praziquantel, ERR: Egg reduction rate.

The rs951840747 (CT) (*p* = 0.01) of *CYP1A2* was significantly associated with PZQ efficacy and had the highest OR, implying that individuals possessing this genotype had a 3.61 (95% CI: 1.30, 10) increase in odds of clearing infection compared to those without this genotype. This was closely followed by the rs1039108105 (GC) genotype (*p* = 0.03) of *CYP3A5*, which also increased the odds of clearing infection with the presence of this SNP by 3.04 (95% CI: 1.08, 8.51). Both rs951840747 and rs1039108105 were only detected as a heterozygous genotype, therefore the interpretation of the dominant, recessive, and Cochran-Armitage trend models was not relevant. A similar effect was observed with rs6976017 (*p* = 0.045) of *CYP3A5*, whose significance via the genotypic association model arose from the rs6976017 (GA) heterozygous genotype, with individuals possessing this genotype over double the odds (OR: 2.19, 95% CI: 1.0, 4.81) of clearing infection compared to those with alternate genotypes at this site. The rs6976017 (AA) homozygous genotype of *CYP3A5* was not found to be significantly associated with PZQ efficacy (*p* = 0.079), which may be attributed to the smaller sample size of the rs6976017 (AA) in comparison. As the rs6976017 (AA) genotype was not a significant finding, the insignificant associations of the allelic, dominant, trend and recessive models were not evaluated with regards to the significance of the presence of rs6976017 (GA) in individuals who cleared infection due to the combinative analysis. Of the 4 SNPs found to be significant, genotypic analysis of rs28371702 of *CYP2D6* was the closest to the significance threshold (*p* = 0.047), yet both the (AC) and (CC) genotype of rs28371702 had a significant association when individually associated with PZQ efficacy. From the analysis of rs28371702 genotype frequencies it appears that the two genotypes had different affinities towards treatment outcome (Figure 3).

The rs28371702 (AC) of *CYP2D6* was significantly associated with increased odds of an individual clearing infection (OR: 2.58, 95% CI: 1.20, 5.55) and the homozygous rs28371702 (CC) was significantly associated with increased odds of an individual not clearing infection (OR: 2.36, 95% CI: 1.11, 5.04). The results of basic associations using a recessive model support these findings, resulting in a significant difference in those individuals who did not clear infection with the rs28371702 (CC) genotype compared to rs28371702 (AA + AC). Due to the conflicting PZQ efficacy per genotype the results of the dominant, allelic, and Cochran-Armitage trend models, which all imply that with an increasing number of alleles there was an increased risk of being significantly associated with the same treatment outcome, were not significant (0.5 < *p* < 0.1).

### 3.3 Combinative Analysis of Single Nucleotide Polymorphisms Associated With Praziquantel Efficacy

#### 3.3.1 Linkage Disequilibrium and LAMPLINK Analysis

We performed conditional and haplotype Linkage Disequilibrium (LD) analyses to assess whether SNPs detected in this study, particularly those SNPs significantly associated with PZQ efficacy, were independent with no secondary associations. LD analysis for each chromosome can be seen in Supplementary Figure S5, and 24 SNP pairs had evidence of LD. Of specific interest was rs6976017 of *CYP3A5*, found to be significantly associated with PZQ efficacy, which had strong evidence of LD with Novel-14 (Supplementary Table S7) and a high LOD of 6.24 and D’ value of 1. This suggested a strong chance that these SNPs are co-inherited, yet the low correlation value (*r* ^2^ = 0.22) and an OR of 0.687 towards clearing infection casts doubt on this association in relation to predicting PZQ efficacy. This indicates co-inheritance but no ability to predict PZQ efficacy based on the shared haplotype in the Zimbabwean subjects, supporting the independence of rs6976017 associations. No other SNPs significantly associated with PZQ efficacy were found to be in LD, indicating single-variant associations rather than a combinative effect of other loci. Haplotype analysis found three haplotype blocks across chromosome 7 and chromosome 10, with the LD statistics of each block displayed in Supplementary Table S8. The frequencies of each haplotype block were analysed for significant differences in PZQ efficacy (Supplementary Table S9), with no haplotype blocks significantly associated. Additionally, no significant SNP combinations were detected using LAMPLINK analysis, with 411 testable combinations assessed and an adjusted significance level of *p* = 1.22 × 10^−4^.

#### 3.3.2 Random Forest Model

To determine which SNPs were most predictive of PZQ efficacy, we assessed SNP importance in determining an individual’s treatment outcome, and whether this could be correctly predicted and classified using a Random Forest (RF) model. RFs allow the assessment of the multiple SNPs in an individual, not their overall frequencies in the whole population, and produced the importance of each SNP based on all the SNPs included. The summary of each RF model and the resulting misclassification rate can be seen in Supplementary Figure S6. The RF model with the lowest error rate was selected, with an overall misclassification rate of 29% (Supplementary Table S10), indicating a misclassification rate below what would be expected by chance. The most important SNPs with regards to predicting PZQ efficacy are presented in Supplementary Figure S6A. To evaluate if the SNPs significantly associated with PZQ efficacy *via* the univariate analysis were the strongest predictors when combined with other SNPs in this model, the RF importance vs. significance was plotted. Supplementary Figure S6B shows the normalised genotypic χ^2^ test of association of SNPs plotted against their permutation importance values from the RF model. There was a consensus between the strength of association and the permutation importance values, with all SNPs significantly associated with PZQ efficacy in the top five most important predictors. Interestingly, rs1022705765, an intron variant located in the *CYP1A2* gene, was not significantly associated with PZQ efficacy but was designated as the second most important predictor. Furthermore, multiple novel SNPs were deemed to be predictive of PZQ efficacy, with upstream *CYP1A2* variant Novel-8 ranking highest in importance at sixth, and the missense *CYP3A4* variant Novel-17 ranking seventh.

## 4 Discussion and Conclusion

To date, there have been minimal studies investigating pharmacogenetics in African populations, especially considering the numerous drugs used on the continent (Radouani et al., 2020). Pharmacogenetics is of growing importance in Africa, with more drugs marketed for use in local populations due to the high disease burden and the increasing management of co-infections requiring concurrent medications. Yet, pharmacogenetic studies in African populations are less abundant than others around the world, as highlighted by a recent review that discovered that only 15 drugs have been clinically evaluated for pharmacogenetic implications in African populations over the vast continent of 54 countries and 1 billion people (Radouani et al., 2020). These studies focused on drugs used in the management of the high disease burden faced by sub-Saharan African populations, focusing on malaria, HIV, and Tuberculosis. Since this review was published, only one study has been conducted that focused on the pharmacogenetics of PZQ and the treatment of schistosomiasis, despite this disease being the second most important parasitic infection on the continent afflicting over 207 million in sub-Saharan Africa (Oyeyemi et al., 2020). The recent paper by Mnkugwe et al. (2021) has presented the first example of pharmacogenetic influences on PZQ concentrations in patients in Tanzania, with CYP2C19*2/*3 genotypes confirmed to increase active PZQ concentrations compared to the CYP2C19*1 and CYP2C19*17. This finding, and the fact that several studies have reported hotspots of persistent schistosome infections with CR lower than 100% across Africa (Sang et al., 2014; Wiegand et al., 2017; Kittur et al., 2019; Knopp et al., 2019; Mawa et al., 2021) only further emphasises the need for research, and particularly pharmacogenetic research, to characterise the determinants of treatment efficacy. As variability in PZQ efficacy has been attributed to multiple host, parasite, and environmental factors, we and others have highlighted the scarcity of studies documenting the impact of PZQ pharmacogenetics in African populations on treatment success (Mduluza and Mutapi, 2017; Zdesenko and Mutapi, 2020). We postulate that some decreased CR can be attributed to the pharmacogenetics of the people treated with PZQ. Consequently, this study aimed to identify polymorphisms in the genes encoding the CYP450 enzymes involved in PZQ metabolism in Zimbabwean participants exposed to *S. haematobium* infection and determined if there was an association with PZQ efficacy.

We identified 70 SNPs across the six CYP450 genes (*CYP1A2*, *CYP2C9*, *CYP2C19*, *CYP2D6*, *CYP3A4*, and *CYP3A5*) and evaluated their minor allele frequency (MAF). Of the detected SNPs, 14.3% were well documented polymorphic CYP alleles and 48.5% had been previously reported on genetic databases but not evaluated in terms of drug metabolism. In fact, 27.2% of SNPs in this Zimbabwean population were not consistent with the MAF previously reported in Africans, with 4.3% also significantly differing from other Zimbabwean frequency studies. This may appear to be a large portion of the SNPs identified, yet these deviations are entirely feasible as the African continent has greater genetic diversity than any other continental population. Furthermore, the 1,000 Genomes study reports the average MAF across the entire continent and therefore is not always representative of the genetic heterogeneity in Zimbabwe (Tishkoff et al., 2009; Rajman et al., 2017). This is especially pivotal when regarding comparisons to MAF in Europeans, as the protocols developed for drug treatments in Europeans e.g., dosage, do not automatically apply to diverse African populations. This study emphasizes the problem in this approach as 87% of SNPs detected here occurred at significantly different frequencies in Africans compared to Europeans, highlighting further problems of standardised drug usage in Zimbabwe based on European studies without the full knowledge of the pharmacogenetic implications. Furthermore, 37.1% of SNPs analysed in this study were novel, and their discovery provides additional insight into the genetic diversity of Zimbabweans and the scarcity of representative genomic information.

To gather further information on the clinical relevance of these SNPs, we used computational tools to predict if these variants shift the metabolic capacity of each enzyme and whether this elicited variability in the efficacy of a PZQ treatment. We identified multiple missense, synonymous, intron and upstream genetic variants that could be damaging to CYP function, but three detected CYP alleles challenged the accuracy of these predictions. Firstly, the CYP2D6*17 polymorphism was predicted to be a tolerated change, with Thr107Ile having no impact on *CYP2D6* function. Yet, CYP2D6*17 is reported to be one of the most functionally important SNP in African populations, resulting in a decrease in *CYP2D6* function (Dandara et al., 2001). This was also the case for the synonymous variant CYP2C19*2, which is reported to be the most frequent *CYP2C19* defect worldwide, reducing the enzymes activity *via* an aberrant splice site (Dandara et al., 2001). Yet again, this change was predicted to be a tolerated change in the Zimbabwean population. Conversely, CYP2C9*9 was predicted to be a deleterious variant, yet CYP2C9*9 has been reported as having no translated impact on drug clearance when investigated *in vivo* (Matimba et al., 2009). These discrepancies in the ability to computationally predict functional effect without an *in vivo* analysis of the SNP phenotype highlights the need for further investigation into the *in vivo* activities of the SNPs detected in this study. This is particularly relevant for the novel SNPs detected in this study. Novel SNPs were found in five of the six CYP450 enzymes evaluated in this study, but two prominent novel missense SNPs are of particular interest due to their amino acid changes; Novel-17 and Novel-19 of *CYP3A4*. Novel-19 was of particular interest as the alteration of the Val393 amino acid in the binding pocket of the *CYP3A4* enzyme (Lill et al., 2006) may have implications on the function of the enzyme. These novel missense changes further emphasise the need for more definitive studies on the functional impact of the novel SNPs.

Multiple SNPs detected in this study had already been reported to be significantly associated with the success or failure of a drug treatment, specifically the CYP1A2*1C (Nakajima et al., 1999), CYP1A2*1F (Lu et al., 2020), CYP1A2*1K (Al-Ahmad et al., 2017) of *CYP1A2*, rs9332127 of *CYP2C9* (Chen et al., 2014), CYP2C19*2 (Dandara et al., 2001) and CYP2C19*17 (Sim et al., 2006; Gawrońska-Szklarz et al., 2012) of *CYP2C19*, CYP2D6*17 (Masimirembwa et al., 1996) and rs28371702 (Zhang et al., 2021) of *CYP2D6*, CYP3A5*3, CYP3A5*6 (Kuehl et al., 2001; Sanghavi et al., 2017), rs4646453 (Wang et al., 2019), and rs6976017 (Jorgensen et al., 2009) of *CYP3A5*. Considering the additional predicted functional effects described in this study, we subsequently determined whether there was an association between each SNP and the efficacy of a PZQ treatment; whether the individual cleared or did not clear infection. Therefore, by testing the statistical association of each SNP to the treatment outcome, we also investigated whether the presence of a SNP could be used to predict treatment success or failure. Our data revealed that four SNPs were significantly associated with PZQ efficacy, with the results driven by SNPs in the *CYP1A2*, *CYP2D6*, and *CYP3A5* genes. From *in vitro* assessments of PZQ metabolism, contributions of these CYPs to the metabolism of PZQ are estimated as *CYP1A2* (39%), *CYP2D6* (<10%), and *CYP3A4/5* (30%) (Masimirembwa et al., 2013). Three of the four SNPs associated with PZQ efficacy were found to significantly increase the odds of an individual clearing infection, including the rs951840747 of *CYP1A2*, and rs1039108105 and rs6976017 of *CYP3A5*. The SNP with the highest OR was rs951840747 (CT) (*p* = 0.01) of *CYP1A2*, signifying that individuals possessing this genotype had a 3.61 increase in odds of clearing infection. The rs951840747 is located on intron 6 of the *CYP1A2* gene and has not been previously reported in the literature in relation to drug metabolism. Conceivably, this functional effect may be a similar mechanism to CYP1A2*1K, also a *CYP1A2* intron SNP, whose presence results in decreased enzyme activity and increased drug concentrations; which for rs951840747 would result in sustained lethal schistosome levels and could contribute to an increased odds of clearing infection (Zimmermann et al., 2007). This is a particularly relevant finding as Nleya and colleagues have provided evidence in Zimbabwean volunteers that *CYP1A2* is the predominant excretion route of PZQ to its main metabolite (Nleya et al., 2019). Therefore, although an individual with this SNP had a significantly increased odds of clearing infection, further pharmacokinetic evaluation is required to confirm if this intron variant contributes towards decreased metabolism of PZQ *via CYP1A2*, preventing the removal of the active parent drug from circulation to its main metabolite and increasing success and efficacy of PZQ treatment.

Decreased metabolism of PZQ may also be representative of the functional effect of the rs1039108105 and rs6976017 variants in the *CYP3A5* gene, which both significantly increased the odds of an individual clearing infection and thus the efficacy of PZQ. *CYP3A4/5* also mediates the biotransformation of PZQ (Wang et al., 2014), so any detrimental alterations to its function can impair metabolism, maintaining exposure of the active drug to the schistosomes and increasing parasite death. There was no literature regarding rs1039108105, but the rs6976017 variant has been sparsely reported. One study significantly associates the SNP with warfarin sensitivity during efficacy studies in a European population (Jorgensen et al., 2009). The pharmacogenetic mechanism described derives from diminished clearance of warfarin, creating increased concentrations than expected from the normal dose. A parallel effect may be occurring with rs6976017 in this study, as there were significantly higher odds of an individual possessing this SNP clearing infection potentially due to increased PZQ concentrations *via* decreased metabolism of *CYP3A5*.

However, the rs28371702 variant of the *CYP2D6* gene had a different affinity to treatment outcome than the other SNPs significantly associated with PZQ efficacy. The homozygous (CC) genotype had over double the odds of an individual with this genotype not clearing infection, whereas the heterozygous (AC) genotype increased the odds of clearing infection by 2.6 times. rs28371702 has previously been reported by to have different *CYP2D6* phenotypes for each genotype, significantly effecting the pharmacokinetics of aripiprazole during a pharmacokinetic-pharmacogenetic study in healthy Chinese subjects (Zhang et al., 2021). However, that study reported the homozygous rs28371702 (CC) genotype was significantly associated with a decreased T_max_ and increased exposure to aripiprazole (indicative of decreased *CYP2D6* function), and the rs28371702 (AC) genotype decreased exposure of aripiprazole (indicative of an ultra-rapid *CYP2D6* function). This contradicts the findings of this study, in which rs28371702 (CC) had significantly more individuals not clearing infection, indicating decreased PZQ exposure and PZQ efficacy. Sustained exposure of the schistosomes to PZQ concentrations above 1 μM for at least 6 h is critical for parasite death the occur (Nleya et al., 2019) and the eventual success of a PZQ treatment, therefore unmaintained lethal PZQ concentrations due to increased *CYP2D6* metabolism will fail to successfully destroy the schistosome load and clear infection. Yet, as *CYP2D6* has a smaller role than other CYPs in the metabolism of PZQ (<10%), the combined influence of SNPs in other CYP pathways with a greater role in PZQ metabolism may be the reasoning behind the discrepancy. Consequently, greater research into the exact mechanism of this SNP is required, particularly with PZQ pharmacokinetic analysis. Overall, for individuals possessing any of the four genotypes that were significantly associated with increased odds of clearing schistosomiasis infection and consequently increased PZQ efficacy, the mechanism potentially stemmed from the decreased function of the *CYP1A2, CYP2D6*, or *CYP3A5* enzymes, permitting increased PZQ concentrations. Increased PZQ concentrations is commonly observed with other CYP polymorphisms, yet high plasma drug exposure may in fact increase the risk of adverse drug reactions (ADRs). PZQ has been known to cause treatment-related side effects including headaches, abdominal pain, and vomiting upon treatment for schistosomiasis (Midzi et al., 2008), and one study concluded that the PZQ-metabolising CYP3A5*3/*6/*7 variants had a significantly higher number of ADRs than those with no defective alleles (Mnkugwe et al., 2021). However, this study found no association with the CYP3A5 alleles and parent PZQ concentrations, it was postulated that increased concentrations of a metabolite was responsible for these adverse events. Therefore, it must be noted that increased or reduced enzyme activity is not always desirable as it increases the risk of ADRs due to altered drug concentrations (Bijl et al., 2008). Thus, we further highlight the importance of characterising SNPs in the genes involved in the metabolism of PZQ in Zimbabweans as alterations in PZQ concentrations may be amplifying the detrimental side effects of treatment.

Yet, drug metabolism and variations in drug efficacy can include multiple genes and numerous polymorphisms (Shastry, 2007), consequently assessing the combinative power of the SNPs was important to gain the complete view of a SNPs importance in predicting treatment success. LAMPLINK and haplotype analysis of SNP combinations towards PZQ efficacy yielded no results, so evaluations of the importance of the SNPs as predictors was conducted *via* a RF model. RF are a promising method of associating polygenic SNPs with an outcome (Botta et al., 2014), and by classifying these SNPs by importance it allows us to discover those most predictive of PZQ efficacy and support the univariate results. When comparing the independent statistical associations to the RF importance values, they were generally concordant with the univariate *p*-values regarding associations to PZQ efficacy, supporting both approaches. However, they did not agree perfectly. Overall, rs6976017 of *CYP3A5* was determined to be the most predictive SNP of PZQ efficacy. Although rs951840747 had the lowest *p*-value and highest odds of clearing infection, it was fifth in predicting PZQ efficacy when analysed as a combinative data set. Those SNPs not adhering to the univariate results were considered to be due to between-SNP interactions, as the RF model considers the combined effect of all the other SNPs in the data set. Although, predictive importance can be skewed by a high MAF, as was observed with rs1479820461 and Novel-23, which both have a MAF >90%. Higher MAF tends to be associated with higher SNP importance in other studies (Boulesteix et al., 2012), therefore these SNPs predictive importance was attributed to model preference of SNPs with higher MAF rather than actual predictive effect. In this regard, the second most important predictor for PZQ efficacy rs1022705765, an intron 6 variant in the *CYP1A2* gene, had a MAF of 16%, consequently its predictive importance may arise from a SNP interaction rather than a high MAF. rs951840747 is also an intron 6 variant in the *CYP1A2* gene and was significantly associated with PZQ efficacy. Nevertheless, no LD was detected with rs1022705765 (or with any other SNP pair), suggesting a different combinative effect between the two relating to PZQ efficacy. However, studies assessing the combinative effect of pharmacogenetic SNPs are rare in African populations, with no literature assessing SNP interactions during a PZQ treatment (Bienczak et al., 2016). Generally, mutations in the CYP genes introns and gene-flanking regions are drastically under-reported, yet altered functional activity due to intron mutations have been discovered in the *CYP1A2, CYP2C19*, *CYP2D6*, and *CYP3A5* genes, with approximately 15 alleles identified (Ingelman-Sundberg and Sim, 2010). CYP3A5*3 in intron 3 (eighth most predictive SNP) results in an inactive *CYP3A5* protein in individuals who carry the homozygous genotype (*3/*3), something that three individuals in this study possessed (Kuehl et al., 2001). All these individuals did not clear infection, however there was no significance due to the small number of homozygous carriers. This predictive importance, in addition to the frequency of unsuccessfully treated homozygous carrier, suggests the SNPs functional implications on PZQ efficacy is larger than expected, but due to the small sample size its effect was not significant *via* statistical associations in this study. Thus, although there were multiple SNPs, novel and otherwise, in the RF model that were not found to be significantly associated to PZQ efficacy, the predictive power of the SNPs presented in this model shows promise in predicting treatment outcome with a 29% misclassification rate. A recent study evaluating the use of RF models on predicting a binary response for medical data sets between the sample sizes of 60–102 showed that the average misclassification rate of this study was within the expected range for human data (25–50%) as produced by Janitza and Hornung (2018). However, the true impact of the independent associations and the combinative effect of these SNPs must be completely evaluated with pharmacokinetic measures, particularly the novel findings, as the association with PZQ efficacy is currently only inferred.

As far as we know, this is the first investigation into the pharmacogenetics of PZQ efficacy in Zimbabwe and the second to be performed in Africa, with no previous studies evaluating the pharmacogenetic impact in relation to the success or failure of a schistosomiasis treatment. Therefore, SNPs detected in this study may influence only part of PZQ pharmacology, with PZQ efficacy potentially compounded by drug-drug interactions, overall health, and other environmental or nongenetic factors (Sorice et al., 2012). Additionally, study limitations such as the relatively small samples size and the uneven distribution of males to females (76: 38) prevents genetic variants found primarily in one sex being significantly detected, as some CYP genes present different expression patterns between genders (Franconi and Campesi, 2014). Likewise, 152 SNPs passed bioinformatic quality control, but due to the lack of power of low frequency SNPs in a small study population MAF <5% were removed from association analysis, as a disease association in one individual would not be possible to confirm. Consequentially, outcome‐associated SNPs may be disregarded. Also, although we assessed the CYP2D6 pathway we did not assess deletions or multiplications of the gene, both of which could impact PZQ concentrations and resultant PZQ efficacy. Moreover, as this was a novel and exploratory analysis our study did not apply multiple testing correction in the statistical analyses which may increase the type II error. We applied a Yates correction to examine significant findings and reduce the risk of false positives, but as this continuity correction can be very stringent with smaller sample sizes we only used these results to further support our significant findings for future validation of these findings. Lastly, and the most prominent limitation, is the necessity for a pharmacokinetic-pharmacogenetic approach to gain stronger evidence of the SNP associations to PZQ efficacy. It was beyond the scope of this study to collect the time-course of samples required to assess the plasma levels of PZQ during of the anthelminthic treatment. Additionally, despite the valuable novel information generated and the practical issues of obtaining pharmacogenetic participant data, this study this was only based on a single cohort of predominantly young individuals. This is a limitation to a pharmacogenetic study and restricts the interpretations of the data produced by this analysis. Hence, this study should be replicated in a second independent Zimbabwean cohort of increased sample size, age range and with the relevant *in vivo* data for further pharmacokinetic analysis to fully capture and inform in the genetic diversity of this country. It is important to stress that the aim of our analysis of these SNPs was not to confirm the metabolising phenotype for these individuals, but to identify if there may be any underlying genetic factors significantly contributing to variable drug efficacy to justify further investigation into the pharmacogenetics of PZQ treatment. The study achieved this aim and future mechanistic studies will be informative of the overall drug-gene interaction.

Overall, while implementing pharmacogenetics poses a massive financial burden on public health-care systems (Tata et al., 2020), eliminating schistosomiasis is reliant in our understanding of these determinants of drug efficacy. African populations possess various unique genetic markers vital to therapeutic drug responses, henceforth the applicability of pharmacogenomics during PZQ treatments will consequently reduce prevalence of treatment failures and ADRs. The SNPs identified in this study are an important source of potentially unique African pharmacogenetic markers, such as the CYP2D6*17, which could also be applied to multiple drug efficacy studies. The importance of African genetic data has already been highlighted in the near-personalised management of HIV patients in sub-Saharan Africa, with reduced doses of efavirenz given to patients with genetic variations in the *CYP2B6* gene (Matimba et al., 2016). Therefore, the contribution of studies such as this highlights the impact pharmacogenetics can have on the efficacy of a drug treatment, and further promotes precision public health to enrich data on the genomics of African populations. We believe the data obtained in this study can be applicable to multiple drug efficacy studies, and the Zimbabwean genomic data can contribute to the growing genetic map of the country. We are currently undertaking a more thorough investigation of the pharmacogenetics-pharmacokinetics of PZQ in Zimbabweans during a schistosomiasis treatment to gather more conclusive evidence and validate the causality of associations determined in this study.

## Data Availability

The datasets presented in this study can be found in online repositories. The names of the repository/repositories and accession number can be found below: https://www.ncbi.nlm.nih.gov/bioproject/PRJNA826901.
