# Supplementary material for: Pharmacogenetics of Praziquantel Metabolism: Evaluating the Cytochrome P450 Genes of Zimbabwean Patients During a Schistosomiasis Treatment
Source: Front Genet. 2022 Jun 8;13:914372. doi: 10.3389/fgene.2022.914372 (PMC9213834; doi:10.3389/fgene.2022.914372)
Supplement: Supplementary file 1 [file DataSheet1.pdf]

Supplementary Procedure 1: Bioinformatic pipeline

A reference FASTA file containing the reference sequences was aligned to the FASTQ files using BWA to each sample. This was then sorted and indexed according to **FILTER1** using joint variant calling, with low quality calls removed via filtering criteria. Method A, aligned against 71 rs-codes with each samples BAM merged and analysed via joint variants calling; Method B, aligned against each CYP's full gene with each samples BAM merged and analysed via joint variants calling. The purpose of using two methods for the variant calling and hard filtering allowed for full gene coverage and obtaining complete genotyping of each individual. The VCF files post-quality control from each method were merged and assigned corresponding meta-data to be moved forward for analysis.

Bioinformatic alignment for variant calling

<table>
<tr><th>Method</th><th>FASTA Reference File</th><th>Variant Calling Method</th><th>Alignment Tool</th><th>Merged VCF –</th></tr>
<tr><td>A</td><td>FASTA-1:
Customised 71 SNP sequences (401 bases) of specific interest to PZQ metabolism from BGI.</td><td rowspan="2">Joint</td><td rowspan="2">BWA</td><td rowspan="2">All SNPs were called and filtered using FILTER1, and then hard filtered via removal of:
MAF<1%
HWE< 1x10^{-4}
Missing Genotype=0%

After quality filtering all SNPs were carried forward</td></tr>
<tr><td>B</td><td>FASTA-2:
CYP1A2, CYP2C9, CYP2C19, CYP2D6, CYP3A4 and CYP3A5 genes for additional CYP alleles and novel discoveries</td></tr>
</table>

The reference files are available as FASTA-1: BGI.fasta and FASTA-2: Gene.fasta. Removed any SNP with HWE<0.0001 to minimise risk of genotyping errors and MAF<1% was removed due to lack of power to detect association with these variants in this sample size.

FILTER1: Codes

a) Ran MappingBWA.py

--produced .pileup and .sortedbam files to run the combined sample analysis

```
import os

# Import read file names

rf = open('read_files.txt', 'r')

samples = []

for line in rf:
    s = line[:-9]
    samples.append(s)

samples = list(set(samples))

print(len(samples))

rf.close()

def MAPPING(ref):
    count=0
    os.system('samtools faidx {}.fasta'.format(ref))
    for sample in samples:
        count=count+1
        if count<1:
            pass
        else:
            print('\n{}\t{}\n'.format(str(count),sample))
```

```
F = '{}_1.fq.gz'.format(sample)
R = '{}_2.fq.gz'.format(sample)
os.system('bwa mem {}.fasta {} {} > /Volumes/GZ/{}_{}.sam'.format(ref, F, R, sample, ref))
os.system('samtools view -F 4 -Sbh {}_{}.sam > {}_{}.bam'.format(sample, ref, sample, ref))
os.system('samtools sort {}_{}.bam > {}_{}_sorted.bam'.format(sample, ref, sample, ref))
os.system('samtools index {}_{}_sorted.bam'.format(sample, ref))

MAPPING('X')
```

b) samtools mpileup -d10000000 -L10000000 -uf X.fasta -b BWAbamset.txt | bcftools call -cv | bgzip -f > BWA_X_Merged.vcf.gz

c) vcfutils.pl varFilter -Q30 -d5 -D10000000 BWA_X_Merged.vcf | bcftools filter -s LowQual -e 'QUAL<200' | bcftools view -f PASS > BWA_X_Merged_Filtered.vcf

Where X is either =

i) **Gene**, NCBI sequences of full gene of each of the six target CYPs:

NC_000015.10:74748845-74756607 Homo sapiens chromosome 15, GRCh38.p13 Primary Assembly,
NC_000010.11:94938658-94990091 Homo sapiens chromosome 10, GRCh38.p13 Primary Assembly,
NC_000010.11:94762681-94855547 Homo sapiens chromosome 10, GRCh38.p13 Primary Assembly,
NC_000022.11:42126499-42130810 Homo sapiens chromosome 22, GRCh38.p13 Primary Assembly,
NC_000007.14:99756967-99784184 Homo sapiens chromosome 7, GRCh38.p13 Primary Assembly,
NC_000007.14:99648194-99679996 Homo sapiens chromosome 7, GRCh38.p13 Primary Assembly

ii) **BGI**, target regions of particular interest relating to common CYP alleles as described by "Worldwide Distribution of Cytochrome P450 Alleles: A Meta-analysis of Population-scale Sequencing Projects":

rs10264272_chr7_99665012-99665412_C
rs1057910_chr10_94981096-94981496_A
rs1065852_chr22_42130492-42130892_G
rs1135822_chr22_42128980-42129380_A
rs1135823_chr22_42128974-42129374_C
rs1135824_chr22_42128842-42129242_T
rs1135840_chr22_42126411-42126811_C
rs114071557_chr10_94938483-94938883_A
rs118203757_chr10_94842679-94843079_G
rs12248560_chr10_94761700-94762100_C
rs12414460_chr10_94942031-94942431_G
rs12720461_chr15_74748810-74749210_C
rs12721629_chr7_99761977-99762377_G
rs138105638_chr7_99766240-99766640_G
rs140278421_chr10_94780374-94780774_G
rs16947_chr22_42127741-42128141_G
rs17878459_chr10_94774965-94775365_G
rs17879685_chr10_94849795-94850195_C
rs17882687_chr10_94762560-94762960_A
rs17884712_chr10_94775289-94775689_G
rs1799853_chr10_94942090-94942490_C
rs192154563_chr10_94852565-94852965_C
rs199523631_chr10_94942054-94942454_C
rs200183364_chr10_94942055-94942455_G
rs201377835_chr22_42129710-42130110_C
rs2069514_chr15_74745679-74746079_G
rs2256871_chr10_94949017-94949417_A
rs2740574_chr7_99784273-99784673_C
rs28365083_chr7_99652413-99652813_G
rs28371685_chr10_94981024-94981424_C
rs28371686_chr10_94981101-94981501_C
rs28371696_chr22_42130515-42130915_C
rs28371706_chr22_42129570-42129970_G
rs28371717_chr22_42128108-42128508_C
rs28371725_chr22_42127603-42128003_C
rs9332239_chr10_94988820-94989220_C
rs28371733_chr22_42126714-42127114_C
rs28371759_chr7_99763803-99764203_A
rs28399504_chr10_94762506-94762906_A
rs35599367_chr7_99768493-99768893_G
rs35742686_chr22_42128041-42128441_C
rs3892097_chr22_42128745-42129145_C
rs41291556_chr10_94775216-94775616_T
rs41303343_chr7_99652570-99652970_T
rs4244285_chr10_94781659-94782059_G
rs4986893_chr10_94780453-94780853_G
rs4986907_chr7_99769604-99770004_C
rs4986908_chr7_99769569-99769969_C
rs4986910_chr7_99760701-99761101_A
rs5030655_chr22_42128883-42129283_C
rs5030656_chr22_42127973-42128373_C
rs5030862_chr22_42130468-42130868_C
rs5030865_chr22_42128833-42129233_C
rs5030867_chr22_42127656-42128056_T
rs55640102_chr10_94852714-94853114_A
rs56165452_chr10_94981097-94981497_T
rs57505750_chr10_94981001-94981401_T
rs59421388_chr22_42127408-42127808_C
rs61736512_chr22_42128932-42129332_C
rs6413438_chr10_94781658-94782058_C
rs67666821_chr7_99757983-99758383_G
rs67784355_chr7_99762006-99762406_G
rs72549346_chr22_42127330-42127730_G
rs72558189_chr10_94942034-94942434_G
rs730882251_chr22_42126547-42126947_G
rs762551_chr15_74749376-74749776_C
rs776746_chr7_99672716-99673116_T
rs781583846_chr10_94988784-94989184_G
rs7900194_chr10_94942109-94942509_G
rs7902257_chr10_94761465-94761865_G
rs9332131_chr10_94949081-94949481_G

Supplementary Table 1: Demographic characteristics of the 114 Zimbabwean subjects. This included the measurements of praziquantel (PZQ) efficacy, and the division of demographics by the treatment outcome.

Characteristics:			Treatment Outcome Group: Cleared Infection (100% CR)	Treatment Outcome Group: Not Cleared Infection (< 100% CR)
**Number of patients** *(male: female)*			57 *(38:19)*	57 *(38:19)*
**Case-control matching** *(counts)*		**Age category**		
		SAC *(6-15 years)*	56	56
		Adult *(≥ 25 years)*	1	1
**Mean age (years)** *(minimum-maximum±SD)*			10.6 *(6-25 ± 2.8)*	11.1 *(6-61 ± 7.08)*
**Mean number of eggs/ml of urine** *(minimum-maximum±SD)*	**Pre-PZQ**		136.3 *(0.3-748 ± 186.9)*	121.7 *(0.5-692.7 ± 171.9*
		**Count of individuals with *n* eggs/ml of urine**		
		1-5	13	13
		6-10	3	3
		11-49	12	12
		50-499	26	25
		> 500	3	4
	**Post-PZQ**		0	24.8 *(0.3-383.7 ± 57.9)*
		**Count of individuals with *n* eggs/ml of urine**		
		1-5	-	30
		6-10		7
		11-49		12
		50-499		8
		> 500		0
**Mean Egg Reduction Rate (%)**			100	79.64

Mean number of eggs based on *Schistosoma haematobium* infection. All subjects were treated with PZQ upon diagnosis. Cure rate (CR) and egg reduction rate (ERR) based on follow-up 6 weeks later for efficacy check. SAC: School aged children, were aged between 6-15 years old, and adults were aged over 25 years old. These samples were collected from the Murewa and Mutoko districts of Zimbabwe.

Supplementary Table 2: Characteristics and frequencies of the 152 single nucleotide polymorphisms (SNPs) detected in the Zimbabwean study population, in addition to the predicted and reported functional changes of each cytochrome P450 (CYP) isoform.

DNA Strand	Enzyme	SNP	Allele	Ref	Alt	MAF (%)	Chromosome Position	Nucleotide Change	Amino Acid Change	SIFT/GWAVA Prediction	Variant Consequence (Region)
1	CYP1A2	rs2069514	CYP1A2*1C	G	A	29.39	74745879 G>A	-3860G>A	-	-/DEL	Upstream Gene
		rs762551	CYP1A2*1F	C	A	53.51	74749576 C>A	-163C>A	-	-/DEL	Intron (Intron 1)
		rs2069526	CYP1A2*1K	T	G	11.84	74749000 T>G	-10+103T>G	-	-/TOL	Intron (Intron 1)
		NOVEL_74747713	Novel	A	G	6.14	74747713 A>G	-1194A>G	-	-/DEL	Upstream Gene
		NOVEL_74747716	Novel	C	T	7.46	74747716 C>T	-1191C>T	-	-/DEL	Upstream Gene
		NOVEL_74747721	Novel	T	C	4.39	74747721 T>C	-1186T>C	-	-/DEL	Upstream Gene
		NOVEL_74747755	Novel	T	A	3.95	74747755 T>A	-1152T>A	-	-/DEL	Upstream Gene
		NOVEL_74747757	Novel	G	A	10.09	74747757 G>A	-1150G>A	-	-/DEL	Upstream Gene
		NOVEL_74747828	Novel	C	T	8.33	74747828 C>T	-1079C>T	-	-/DEL	Upstream Gene
		NOVEL_74753479	Novel	C	T	3.51	74753479 C>T	1253+209C>T	-	-/TOL	Intron (Intron 6)
		NOVEL_74753482	Novel	G	A	12.72	74753482 G>A	1253+212G>A	-	-/TOL	Intron (Intron 6)
		NOVEL_74753485	Novel	G	C,T	86.84	74753485 G>C,T	1253+215G>C	-	-/TOL	Intron (Intron 6)
		NOVEL_74753489	Novel	G	A	1.32	74753489 G>A	1253+219G>A	-	-/TOL	Intron (Intron 6)
		NOVEL_74753490	Novel	G	A	3.51	74753490 G>A	1253+220G>A	-	-/TOL	Intron (Intron 6)
		NOVEL_74753507	Novel	G	A	1.32	74753507 G>A	1253+237G>A	-	-/TOL	Intron (Intron 6)
		NOVEL_74753510	Novel	C	T	3.51	74753510 C>T	1253+240C>T	-	-/TOL	Intron (Intron 6)
		NOVEL_74753512	Novel	C	T	13.16	74753512 C>T	1253+242C>T	-	-/TOL	Intron (Intron 6)
		NOVEL_74753515	Novel	G	A	7.02	74753515 G>A	1253+245G>A	-	-/TOL	Intron (Intron 6)
		NOVEL_74753521	Novel	A	G	1.32	74753521 A>G	1253+251A>G	-	-/TOL	Intron (Intron 6)
		NOVEL_74755865	Novel	G	A	4.39	74755865 G>A	*777G>A	-	-/DEL	3' UTR (Exon 7)
		rs1022705765	rs1022705765	G	A	10.53	74753493 G>A	1253+223G>A	-	-/TOL	Intron (Intron 6)
		rs1450415112	rs1450415112	A	G	9.65	74753511 A>G	1253+241A>G	-	-/TOL	Intron (Intron 6)
		rs17861150	rs17861150	G	A	2.63	74749561 G>A	-9-169G>A	-	-/DEL	Intron (Intron 1)
		rs17861152	rs17861152	C	G	1.75	74749791 C>G	53C>G	Ser18Cys	TOL/DEL	Missense (Exon 2)
		rs45607039	rs45607039	G	A	3.07	74749234 G>A	-10+337G>A	-	-/TOL	Intron (Intron 1)
		rs951840747	rs951840747	C	T	10.09	74753480 C>T	1253+210C>T	-	-/TOL	Intron (Intron 6)
	CYP2C19	rs12248560	CYP2C19*17	C	T	15.79	94761900 C>T	-806C>T	-	-/DEL	Upstream Gene
		rs7902257	CYP2C19*27	G	A	13.60	94761665 G>A	-1041G>A	-	-/-	Upstream Gene

	rs17884712	CYP2C19*9	G	A	3.07	94775489 G>A	431G>A	Arg144His	DEL/DEL	Missense (Exon 3)
	NOVEL_94779010	Novel	G	A	6.58	94779010 G>A	482-1489G>A	-	-/TOL	Intron (Intron 3)
	NOVEL_94779787	Novel	T	C	3.07	94779787 T>C	482-712T>C	-	-/TOL	Intron (Intron 3)
	NOVEL_94818597	Novel	G	A	1.75	94818597 G>A	820-1899G>A	-	-/TOL	Intron (Intron 5)
	rs1003871436	rs1003871436	G	A	4.39	94779034 G>A	482-1465G>A	-	-/TOL	Intron (Intron 3)
	rs11568729	rs11568729	C	T	2.63	94761923 C>T	-783C>T	-	-/DEL	Upstream Gene
	rs1348635965	rs1348635965	C	T	3.07	94779809 C>T	482-690C>T	-	-/TOL	Intron (Intron 3)
	rs17879992	rs17879992	T	C	11.84	94775871 T>C	481+332T>C	-	-/TOL	Intron (Intron 3)
	rs17882572	rs17882572	G	T	1.75	94850299 G>T	1291+241G>T	-	-/TOL	Intron (Intron 8)
	rs17884938	rs17884938	T	A	9.21	94780934 T>A	642+275T>A	-	-/TOL	Intron (Intron 4)
	rs17885567	rs17885567	C	T	5.70	94850227 C>T	1291+169C>T	-	-/TOL	Intron (Intron 8)
	rs4244285	CYP2C19*2	G	A	16.23	94781859 G>A	681G>A	Pro218	-/TOL	Synonymous (Exon 5)
	rs4917623	rs4917623	T	C	10.96	94849811 T>C	1150-106T>C	-	-/TOL	Intron (Intron 7)
	rs4986894	rs4986894	T	C	14.91	94762608 T>C	-98T>C	-	-/DEL	Upstream Gene
	rs76267522	rs76267522	T	C	5.26	94780959 T>C	642+300T>C	-	-/TOL	Intron (Intron 4)
	rs2256871	CYP2C9*9	A	G	14.04	94949217 A>G	752A>G	His251Arg	DEL/DEL	Missense (Exon 5)
	NOVEL_94956739	Novel	A	G	2.19	94956739 A>G	819+7455A>G	-	-/TOL	Intron (Intron 5)
	NOVEL_94956743	Novel	T	C	7.46	94956743 T>C	819+7459T>C	-	-/TOL	Intron (Intron 5)
	NOVEL_94956762	Novel	C	T	4.39	94956762 C>T	819+7478C>T	-	-/TOL	Intron (Intron 5)
	NOVEL_94977838	Novel	C	T	6.14	94977838 C>T	962-3345C>T	-	-/TOL	Intron (Intron 6)
	rs150488420	rs150488420	A	C	1.32	94948886 A>C	643-222A>C	-	-/TOL	Intron (Intron 4)
	rs1934967	rs1934967	C	T	3.95	94981669 C>T	1149+299C>T	-	-/TOL	Intron (Intron 7)
	rs2017319	rs2017319	C	T	13.60	94988878 C>T	1323C>T	Ala441 (3D)	-/TOL	Synonymous (Exon 9)
CYP2C9	rs2298037	rs2298037	C	T	1.75	94986321 C>T	1291+147C>T	-	-/TOL	Intron (Intron 8)
	rs28371675	rs28371675	C	T	2.63	94942580 C>T	481+239C>T	-	-/TOL	Intron (Intron 3)
	rs75541073	rs75541073	G	A	16.23	94948233 G>A	642+294G>A	-	-/TOL	Intron (Intron 4)
	rs762831584	rs762831584	G	A	1.75	94948193 G>A	642+254G>A	-	-/TOL	Intron (Intron 4)
	rs9332127	rs9332127	G	C	14.47	94947714 G>C	482-65G>C	-	-/TOL	Intron (Intron 3)
	rs9332230	rs9332230	A	T	1.75	94986227 A>T	1291+53A>T	-	-/TOL	Intron (Intron 8)
	rs9332232	rs9332232	T	C	13.60	94986275 T>C	1291+101T>C	-	-/TOL	Intron (Intron 8)
	rs9332238	rs9332238	G	A	1.32	94988735 G>A	1292-112G>A	-	-/TOL	Intron (Intron 8)
	rs9332241	rs9332241	C	T	7.02	94989116 C>T	*88C>T	-	-/TOL	3' UTR (Exon 9)

		rs996837814	rs996837814	C	T	1.32	94942487 C>T	481+146C>T	-	-/TOL	Intron (Intron 3)
		COSV62244324	COSV62244324	T	G	1.32	42128283 T>G	734A>C	Lys245Thr	DEL/-	Missense (Exon 5)
		rs28371706	CYP2D6*17	G	A	16.67	42129770 G>A	320C>T	Thr107Ile	TOL/-	Missense (Exon 2)
		rs3892097	CYP2D6*4	C	T	3.51	42128945 C>T	506-1G>A	-	-/-	Splice Acceptor (Intron 3)
		NOVEL_42128405	Novel	C	A	2.19	42128405 C>A	667-55G>T	-	-/-	Intron (Intron 4)
		rs141824015	rs141824015	T	G	1.32	42127605 T>G	1015A>C	Ile339Leu	DEL/-	Missense (Exon 7)
		rs17002853	rs17002853	A	G	5.70	42128325 A>G	692T>C	Leu231Pro	DEL/-	Missense (Exon 5)
		rs184086520	rs184086520	A	G	1.32	42129436 A>G	353-251T>C	-	-/-	Intron (Intron 2)
		rs184517596	rs184517596	C	T	3.07	42128513 C>T	667-163G>A	-	-/-	Intron (Intron 4)
	CYP2D6	rs28371702	rs28371702	A	C	75.88	42129950 A>C	181-41T>G	-	-/-	Intron (Intron 1)
		rs377590656	rs377590656	G	A	2.63	42126758 G>A	1316-6C>T	-	-/-	Splice Region, Intron (Intron 8)
		rs61736514	rs61736514	G	C	2.63	42127944 G>C	883C>G	Leu295Val	DEL/-	Missense (Exon 6)
		rs746582641	rs746582641	G	A	2.63	42127474 G>A	1146C>T	Ile382	-/-	Synonymous (Exon 7)
		rs771097393	rs771097393	T	C	1.32	42126756 T>C	1316-4A>G	-	-/-	Splice Region, Intron (Intron 8)
		rs77593160	rs77593160	C	T	4.82	42128380 C>T	667-30G>A	-	-/-	Intron (Intron 4)
		rs79331140	rs79331140	G	C	4.39	42128367 G>C	667-17C>G	-	-/-	Intron (Intron 4)
-1		rs866129176	rs866129176	C	G	1.32	42130211 C>G	181-302G>C	-	-/-	Intron (Intron 1)
		NOVEL_99758776	Novel	A	G	1.32	99758776 A>G	1510-548T>C	-	-/TOL	Intron (Intron 13)
		NOVEL_99761730	Novel	A	G	2.19	99761730 A>G	1346+46T>C	-	-/TOL	Intron (Intron 12)
		NOVEL_99761942	Novel	T	C	7.89	99761942 T>C	1254-74A>G	-	-/TOL	Intron (Intron 11)
		NOVEL_99762108	Novel	T	C	16.23	99762108 T>C	1186A>G	Ile396Val	TOL/DEL	Missense (Exon 11)
		NOVEL_99762115	Novel	C	T	7.46	99762115 C>T	1179G>A	Val393	-/TOL	Synonymous (Exon 11)
		NOVEL_99762116	Novel	A	G	7.46	99762116 A>G	1178T>C	Val393Ala	TOL/DEL	Missense (Exon 11)
		NOVEL_99764034	Novel	C	T	16.23	99764034 C>T	866-19G>A	-	-/TOL	Intron (Intron 9)
	CYP3A4	NOVEL_99764099	Novel	C	T	8.33	99764099 C>T	866-84G>A	-	-/TOL	Intron (Intron 9)
		NOVEL_99764101	Novel	T	C	7.89	99764101 T>C	866-86A>G	-	-/TOL	Intron (Intron 9)
		NOVEL_99764156	Novel	T	C	4.39	99764156 T>C	866-141A>G	-	-/TOL	Intron (Intron 9)
		NOVEL_99766206	Novel	G	A	1.75	99766206 G>A	865+171C>T	-	-/TOL	Intron (Intron 9)
		NOVEL_99766252	Novel	C	T	5.26	99766252 C>T	865+125G>A	-	-/TOL	Intron (Intron 9)
		NOVEL_99769333	Novel	C	G	3.95	99769333 C>G	521+435G>C	-	-/TOL	Intron (Intron 6)
		NOVEL_99783700	Novel	C	T	2.19	99783700 C>T	71+311G>A	-	-/DEL	Intron (Intron 1)
		NOVEL_99783721	Novel	G	A	3.51	99783721 G>A	71+290C>T	-	-/DEL	Intron (Intron 1)

	NOVEL_99783752	Novel	G	A	2.19	99783752 G>A	71+259C>T	-	-/DEL	Intron (Intron 1)
	NOVEL_99784449	Novel	A	G	15.35	99784449 A>G	-368T>C	-	-/DEL	Upstream Gene
	NOVEL_99784471	Novel	T	A	11.40	99784471 T>A	-390A>T	-	-/DEL	Upstream Gene
	rs1006181087	rs1006181087	C	T	8.33	99762118 C>T	1176G>A	Val392	-/TOL	Synonymous (Exon 11)
	rs113655375	rs113655375	G	C	3.07	99768818 G>C	522-316C>G	-	-/TOL	Intron (Intron 6)
	rs12721622	rs12721622	A	T	9.65	99768236 A>T	670+118T>A	-	-/TOL	Intron (Intron 7)
	rs139943057	rs139943057	T	C	1.32	99758944 T>C	1510-716A>G	-	-/TOL	Intron (Intron 13)
	rs141699118	rs141699118	T	G	1.75	99758514 T>G	1510-286A>C	-	-/TOL	Intron (Intron 13)
	rs143997373	rs143997373	A	G	2.19	99768893 A>G	522-391T>C	-	-/TOL	Intron (Intron 6)
	rs144721069	rs144721069	A	T	3.07	99784296 A>T	-215T>A	-	-/DEL	Upstream Gene
	rs1479820461	rs1479820461	G	C	99.12	99783699 G>C	71+312C>G	-	-/DEL	Intron (Intron 1)
	rs148274077	rs148274077	G	T	1.32	99769506 G>T	521+262C>A	-	-/TOL	Intron (Intron 6)
	rs149507710	rs149507710	A	G	3.95	99768850 A>G	522-348T>C	-	-/TOL	Intron (Intron 6)
	rs2687110	rs2687110	A	T	62.28	99773128 A>T	219-439T>A	-	-/TOL	Intron (Intron 3)
	rs2687116	rs2687116	C	A	23.25	99768320 C>A	670+34G>T	-	-/TOL	Intron (Intron 7)
	rs28371758	rs28371758	G	A	3.07	99766781 G>A	799-338C>T	-	-/TOL	Intron (Intron 8)
	rs28988583	rs28988583	A	G	11.84	99769086 A>G	522-584T>C	-	-/TOL	Intron (Intron 6)
	rs28988584	rs28988584	A	C	3.07	99768269 A>C	670+85T>G	-	-/TOL	Intron (Intron 7)
	rs28988587	rs28988587	A	G	2.63	99766779 A>G	799-336T>C	-	-/TOL	Intron (Intron 8)
	rs28988588	rs28988588	A	C	2.63	99766637 A>C	799-194T>G	-	-/TOL	Intron (Intron 8)
	rs28988601	rs28988601	A	C	2.19	99758814 A>C	1510-586T>G	-	-/TOL	Intron (Intron 13)
	rs28988602	rs28988602	T	G	1.32	99758565 T>G	1510-337A>C	-	-/TOL	Intron (Intron 13)
	rs34309648	rs34309648	G	A	2.63	99766137 G>A	865+240C>T	-	-/TOL	Intron (Intron 9)
	rs35073169	rs35073169	G	T	3.07	99759062 G>T	1510-834C>A	-	-/TOL	Intron (Intron 13)
	rs3735451	rs3735451	T	C	82.02	99758352 T>C	1510-124A>G	-	-/TOL	Intron (Intron 13)
	rs4986911	rs4986911	C	G	2.19	99763764 C>G	1026+91G>C	-	-/TOL	Intron (Intron 10)
	rs746971934	rs746971934	C	A	8.33	99762120 C>A	1174G>T	Val392Leu	DEL/DEL	Missense (Exon 11)
	rs759741768	rs759741768	G	C	4.39	99764149 G>C	866-134C>G	-	-/TOL	Intron (Intron 9)
	rs778270963	rs778270963	C	A	15.79	99762101 C>A	1193G>T	Ser398Ile	TOL/-	Missense (Exon 11)
	rs915268104	rs915268104	C	T	6.58	99783718 C>T	71+293G>A	-	-/DEL	Intron (Intron 1)
CYP3A5	rs776746	CYP3A5*3	T	C	15.79	99672916 T>C	219-237A>G	-	-/-	Intron (Intron 3)
	rs10264272	CYP3A5*6	C	T	15.35	99665212 C>T	624G>A	Lys208	-/DEL	Synonymous (Exon 7)

NOVEL_99656073	Novel	C	A	2.19	99656073 C>A	1027-3294G>T	-	-/TOL	Intron (Intron 10)
NOVEL_99656077	Novel	C	A	1.75	99656077 C>A	1027-3298G>T	-	-/TOL	Intron (Intron 10)
NOVEL_99666556	Novel	A	G	6.14	99666556 A>G	521+45T>C	-	-/DEL	Intron (Intron 6)
NOVEL_99672192	Novel	C	T	3.51	99672192 C>T	318+388G>A	-	-/TOL	Intron (Intron 4)
NOVEL_99672211	Novel	G	C,T	97.81	99672211 G>C,T	318+369C>G	-	-/TOL	Intron (Intron 4)
NOVEL_99672248	Novel	C	T	3.07	99672248 C>T	318+332G>A	-	-/TOL	Intron (Intron 4)
NOVEL_99672251	Novel	C	T	5.26	99672251 C>T	318+329G>A	-	-/TOL	Intron (Intron 4)
NOVEL_99672254	Novel	G	A	15.79	99672254 G>A	318+326C>T	-	-/TOL	Intron (Intron 4)
NOVEL_99672274	Novel	A	T	3.07	99672274 A>T	318+306T>A	-	-/TOL	Intron (Intron 4)
NOVEL_99672285	Novel	C	T	15.35	99672285 C>T	318+295G>A	-	-/TOL	Intron (Intron 4)
rs1039108105	rs1039108105	G	C	9.21	99656082 G>C	1027-3303C>G	-	-/TOL	Intron (Intron 10)
rs1361335502	rs1361335502	G	A	2.63	99672286 G>A	318+294C>T	-	-/DEL	Intron (Intron 4)
rs140259921	rs140259921	A	C	1.75	99662547 A>C	865+269T>G	-	-/TOL	Intron (Intron 9)
rs142823108	rs142823108	A	G	1.75	99666676 A>G	446T>C	Ile149Thr	DEL/DEL	Missense (Exon 6)
rs145025512	rs145025512	T	C	1.32	99660415 T>C	1026+84A>G	-	-/TOL	Intron (Intron 10)
rs1458424958	rs1458424958	A	G	5.26	99673203 A>G	219-524T>C	-	-/TOL	Intron (Intron 3)
rs1462057054	rs1462057054	A	G	16.67	99672249 A>G	318+331T>C	-	-/TOL	Intron (Intron 4)
rs2040992	rs2040992	G	A	100.00	99664949 G>A	670+217C>T	-	-/DEL	Intron (Intron 7)
rs28365069	rs28365069	A	G	2.19	99648403 A>G	1414-3T>C	-	-/DEL	Splice Region, Intron (Intron 12)
rs28365086	rs28365086	C	T	4.82	99663111 C>T	799-229G>A	-	-/DEL	Intron (Intron 8)
rs28383471	rs28383471	G	C	1.32	99666768 G>C	433-79C>G	-	-/DEL	Intron (Intron 5)
rs28383472	rs28383472	T	C	3.07	99665182 T>C	654A>G	Pro227	-/-	Synonymous (Exon 7)
rs41303322	rs41303322	T	C	10.53	99664999 T>C	670+167A>G	-	-/TOL	Intron (Intron 7)
rs4646453	rs4646453	C	A	9.21	99662739 C>A	865+77G>T	-	-/TOL	Intron (Intron 9)
rs68178885	rs68178885	A	G	1.32	99666729 A>G	433-40T>C	-	-/DEL	Intron (Intron 5)
rs6976017	rs6976017	G	A	20.18	99652376 G>A	1253+177C>T	-	-/TOL	Intron (Intron 11)
rs8175345	rs8175345	G	A	9.65	99672695 G>A	219-16C>T	-	-/DEL	Intron (Intron 3)
rs942147254	rs942147254	T	C	4.39	99656081 T>C	1027-3302A>G	-		Intron (Intron 10)

SIFT: Sorting Intolerant From Tolerant, GWAVA: Genome-Wide Annotation of Variants. (*) represents a significant difference in the minor allele frequency (MAF) in this Zimbabwean study population compared to the reported MAF in the African population in the ALFA study from dbSNP. DEL: Deleterious effect on protein function, TOL: Tolerated effect on protein function

Supplementary Table 3: The strength of LD based on the following test statistics: LOD, D', D' 95% CIs, and r^2. Two SNPs were determined to be in complete LD where the LOD score ≥3, the D'=1, 95% CIs of the D' value between them has a lower limit ≥ 0.7 and an upper limit ≥ 0.98, and with association measured using with $r^2 = 1$

Evidence of LD	D'	LOD	r^2	CI (95%)
COMPLETE	D' = 1	LOD ≥3	$r^2 = 1$	CI (95%) [≥0.7, ≥0.98]
STRONG	D' = 1	LOD ≥3	≥ 0.8	CI (95%) [≥0.7, ≥0.98]
INTERMEDIATE	D' = 1	LOD ≥3	< 0.8	CI (95%) [≥0.7, ≥0.98]

D' is the value of D prime between the two loci; LOD is the log of the likelihood odds ratio, a measure of confidence; r^2 is the correlation coefficient between the two loci; CI (95%) is 95% confidence interval bounds on D'.

Supplementary Table 4: Tests of association between 70 detected single nucleotide polymorphisms (SNPs) with a minor allele frequency (MAF) > 5% and the outcome of a praziquantel (PZQ) treatment using the genetic models available in PLINK.

		Genotypic Association χ2 test					Allelic Association χ2 test					Cochran-Armitage Trend T^2 test					Dominant Model χ2 test					Recessive Model χ2 test				
Enzyme	SNP ID	χ2	*DF*	*P*	NC	C	χ2	*DF*	*P*	NC	C	χ2	*DF*	*P*	NC	C	χ2	*DF*	*P*	NC	C	χ2	*DF*	*P*	NC	C
CYP1A2	CYP1A2*1C	2.209	2	0.331	9/19/29	4/22/31	1.036	1	0.309	37/77	30/84	0.914	1	0.339	37/77	30/84	0.141	1	0.708	28/29	26/31	2.171	1	0.141	9/48	4/53
	CYP1A2*1F	0.763	2	0.683	19/26/12	17/24/16	0.635	1	0.426	64/50	58/56	0.568	1	0.451	64/50	58/56	0.758	1	0.384	45/12	41/16	0.162	1	0.687	19/38	17/40
	CYP1A2*1K	2.681	2	0.262	1/8/48	1/15/41	2.059	1	0.151	10/104	17/97	1.991	1	0.158	10/104	17/97	2.511	1	0.113	9/48	16/41	0.000	1	1.000	1/56	1/56
	NOVEL_74747713	2.931	1	0.087	0/10/47	0/4/53	2.740	1	0.098	10/104	4/110	2.931	1	0.087	10/104	4/110	2.931	1	0.087	10/47	4/53	NA	NA	NA	0/57	0/57
	NOVEL_74747716	0.622	1	0.430	0/7/50	0/10/47	0.572	1	0.449	7/107	10/104	0.622	1	0.430	7/107	10/104	0.622	1	0.430	7/50	10/47	NA	NA	NA	0/57	0/57
	NOVEL_74747757	1.362	1	0.243	0/14/43	0/9/48	1.209	1	0.272	14/100	9/105	1.362	1	0.243	14/100	9/105	1.362	1	0.243	14/43	9/48	NA	NA	NA	0/57	0/57
	NOVEL_74747828	0.063	1	0.802	0/10/47	0/9/48	0.057	1	0.811	10/104	9/105	0.063	1	0.802	10/104	9/105	0.063	1	0.802	10/47	9/48	NA	NA	NA	0/57	0/57
	NOVEL_74753482	0.046	1	0.830	0/15/42	0/14/43	0.040	1	0.842	15/99	14/100	0.046	1	0.830	15/99	14/100	0.046	1	0.830	15/42	14/43	NA	NA	NA	0/57	0/57
	NOVEL_74753485	2.895	1	0.089	46/11/0	38/19/0	2.457	1	0.117	103/11	95/19	2.895	1	0.089	103/11	95/19	NA	NA	NA	57/0	57/0	2.895	1	0.089	46/11	38/19
	NOVEL_74753512	0.000	1	1.000	0/15/42	0/15/42	0.000	1	1.000	15/99	15/99	0.000	1	1.000	15/99	15/99	0.000	1	1.000	15/42	15/42	NA	NA	NA	0/57	0/57
	NOVEL_74753515	0.291	1	0.590	0/9/48	0/7/50	0.269	1	0.604	9/105	7/107	0.291	1	0.590	9/105	7/107	0.291	1	0.590	9/48	7/50	NA	NA	NA	0/57	0/57
	rs1022705765	1.900	1	0.168	0/15/42	0/9/48	1.676	1	0.195	15/99	9/105	1.900	1	0.168	15/99	9/105	1.900	1	0.168	15/42	9/48	NA	NA	NA	0/57	0/57
	rs1450415112	2.028	1	0.155	0/8/49	0/14/43	1.811	1	0.178	8/106	14/100	2.028	1	0.155	8/106	14/100	2.028	1	0.155	8/49	14/43	NA	NA	NA	0/57	0/57
	rs951840747	6.591	1	0.010	0/6/51	0/17/40	5.851	1	0.016	6/108	17/97	6.591	1	0.010	6/108	17/97	6.591	1	0.010	6/51	17/40	NA	NA	NA	0/57	0/57
CYP2C9	CYP2C9*9	0.000	2	1.000	1/14/42	1/14/42	0.000	1	1.000	16/98	16/98	0.000	1	1.000	16/98	16/98	0.000	1	1.000	15/42	15/42	0.000	1	1.000	1/56	1/56
	NOVEL_94956743	0.069	1	0.793	0/9/48	0/8/49	0.064	1	0.801	9/105	8/106	0.069	1	0.793	9/105	8/106	0.069	1	0.793	9/48	8/49	NA	NA	NA	0/57	0/57
	NOVEL_94977838	0.326	1	0.568	0/6/51	0/8/49	0.304	1	0.581	6/108	8/106	0.326	1	0.568	6/108	8/106	0.326	1	0.568	6/51	8/49	NA	NA	NA	0/57	0/57
	rs2017319	0.420	2	0.811	2/13/42	1/12/44	0.336	1	0.562	17/97	14/100	0.315	1	0.575	17/97	14/100	0.189	1	0.663	15/42	13/44	0.342	1	0.559	2/55	1/56
	rs75541073	0.674	2	0.714	2/14/41	1/17/39	0.032	1	0.858	18/96	19/95	0.032	1	0.857	18/96	19/95	0.168	1	0.682	16/41	18/39	0.342	1	0.559	2/55	1/56
	rs9332127	0.419	2	0.811	1/13/43	1/16/40	0.319	1	0.572	15/99	18/96	0.328	1	0.567	15/99	18/96	0.399	1	0.528	14/43	17/40	0.000	1	1.000	1/56	1/56
	rs9332232	0.420	2	0.811	2/13/42	1/12/44	0.336	1	0.562	17/97	14/100	0.315	1	0.575	17/97	14/100	0.189	1	0.663	15/42	13/44	0.342	1	0.559	2/55	1/56
	rs9332241	1.493	2	0.474	1/8/48	1/4/52	1.075	1	0.300	10/104	6/108	0.901	1	0.343	10/104	6/108	1.303	1	0.254	9/48	5/52	0.000	1	1.000	1/56	1/56
CYP2C19	CYP2C19*17	1.929	2	0.381	3/9/45	3/15/39	1.188	1	0.276	15/99	21/93	0.983	1	0.322	15/99	21/93	1.629	1	0.202	12/45	18/39	0.000	1	1.000	3/54	3/54
	CYP2C19*27	0.044	1	0.833	0/16/41	0/15/42	0.037	1	0.847	16/98	15/99	0.044	1	0.833	16/98	15/99	0.044	1	0.833	16/41	15/42	NA	NA	NA	0/57	0/57
	NOVEL_94779010	0.691	1	0.406	0/6/51	0/9/48	0.642	1	0.423	6/108	9/105	0.691	1	0.406	6/108	9/105	0.691	1	0.406	6/51	9/48	NA	NA	NA	0/57	0/57
	rs17879992	1.591	2	0.452	2/12/43	2/7/48	1.050	1	0.305	16/98	11/103	0.874	1	0.350	16/98	11/103	1.362	1	0.243	14/43	9/48	0.000	1	1.000	2/55	2/55
	rs17884938	1.053	2	0.591	1/9/47	0/10/47	0.052	1	0.819	11/103	10/104	0.052	1	0.819	11/103	10/104	0.000	1	1.000	10/47	10/47	1.009	1	0.315	1/56	0/57

	rs17885567	0.087	1	0.768	0/6/51	0/7/50	0.082	1	0.775	6/108	7/107	0.087	1	0.768	6/108	7/107	0.087	1	0.768	6/51	7/50	NA	NA	NA	0/57	0/57
	rs4244285	1.047	2	0.593	1/15/41	3/14/40	0.290	1	0.590	17/97	20/94	0.273	1	0.602	17/97	20/94	0.043	1	0.836	16/41	17/40	1.036	1	0.309	1/56	3/54
	rs4917623	0.429	2	0.807	1/9/47	2/10/45	0.404	1	0.525	11/103	14/100	0.353	1	0.553	11/103	14/100	0.225	1	0.635	10/47	12/45	0.342	1	0.559	1/56	2/55
	rs4986894	0.182	2	0.913	1/14/42	1/16/40	0.138	1	0.710	16/98	18/96	0.144	1	0.705	16/98	18/96	0.174	1	0.677	15/42	17/40	0.000	1	1.000	1/56	1/56
	rs76267522	0.373	1	0.542	0/7/50	0/5/52	0.352	1	0.553	7/107	5/109	0.373	1	0.542	7/107	5/109	0.373	1	0.542	7/50	5/52	NA	NA	NA	0/57	0/57
	CYP2D6*17	3.260	2	0.196	5/14/38	1/12/44	3.158	1	0.076	24/90	14/100	2.679	1	0.102	24/90	14/100	1.564	1	0.211	19/38	13/44	2.815	1	0.093	5/52	1/56
CYP2D6	rs17002853	0.781	1	0.377	0/5/52	0/8/49	0.734	1	0.392	5/109	8/106	0.781	1	0.377	5/109	8/106	0.781	1	0.377	5/52	8/49	NA	NA	NA	0/57	0/57
	rs28371702	6.105	2	0.047	37/18/2	25/31/1	2.899	1	0.089	92/22	81/33	3.511	1	0.061	92/22	81/33	0.342	1	0.559	55/2	56/1	5.092	1	0.024	37/20	25/32
	NOVEL_99761942	2.375	1	0.123	0/6/51	0/12/45	2.171	1	0.141	6/108	12/102	2.375	1	0.123	6/108	12/102	2.375	1	0.123	6/51	12/45	NA	NA	NA	0/57	0/57
	NOVEL_99762108	3.241	1	0.072	0/14/43	0/23/34	2.613	1	0.106	14/100	23/91	3.241	1	0.072	14/100	23/91	3.241	1	0.072	14/43	23/34	NA	NA	NA	0/57	0/57
	NOVEL_99762115	1.728	1	0.189	0/6/51	0/11/46	1.589	1	0.208	6/108	11/103	1.728	1	0.189	6/108	11/103	1.728	1	0.189	6/51	11/46	NA	NA	NA	0/57	0/57
	NOVEL_99762116	1.728	1	0.189	0/6/51	0/11/46	1.589	1	0.208	6/108	11/103	1.728	1	0.189	6/108	11/103	1.728	1	0.189	6/51	11/46	NA	NA	NA	0/57	0/57
	NOVEL_99764034	1.961	1	0.161	0/15/42	0/22/35	1.581	1	0.209	15/99	22/92	1.961	1	0.161	15/99	22/92	1.961	1	0.161	15/42	22/35	NA	NA	NA	0/57	0/57
	NOVEL_99764099	0.063	1	0.802	0/10/47	0/9/48	0.057	1	0.811	10/104	9/105	0.063	1	0.802	10/104	9/105	0.063	1	0.802	10/47	9/48	NA	NA	NA	0/57	0/57
	NOVEL_99764101	0.264	1	0.608	0/10/47	0/8/49	0.241	1	0.623	10/104	8/106	0.264	1	0.608	10/104	8/106	0.264	1	0.608	10/47	8/49	NA	NA	NA	0/57	0/57
	NOVEL_99766252	0.373	1	0.542	0/7/50	0/5/52	0.352	1	0.553	7/107	5/109	0.373	1	0.542	7/107	5/109	0.373	1	0.542	7/50	5/52	NA	NA	NA	0/57	0/57
	NOVEL_99784449	0.041	1	0.839	0/18/39	0/17/40	0.034	1	0.854	18/96	17/97	0.041	1	0.839	18/96	17/97	0.041	1	0.839	18/39	17/40	NA	NA	NA	0/57	0/57
	NOVEL_99784471	0.199	1	0.655	0/12/45	0/14/43	0.174	1	0.677	12/102	14/100	0.199	1	0.655	12/102	14/100	0.199	1	0.655	12/45	14/43	NA	NA	NA	0/57	0/57
CYP3A4	rs1006181087	1.579	1	0.209	0/7/50	0/12/45	1.435	1	0.231	7/107	12/102	1.579	1	0.209	7/107	12/102	1.579	1	0.209	7/50	12/45	NA	NA	NA	0/57	0/57
	rs12721622	0.225	1	0.635	0/10/47	0/12/45	0.201	1	0.654	10/104	12/102	0.225	1	0.635	10/104	12/102	0.225	1	0.635	10/47	12/45	NA	NA	NA	0/57	0/57
	rs1479820461	2.036	1	0.154	55/2/0	57/0/0	2.018	1	0.156	112/2	114/0	2.036	1	0.154	112/2	114/0	NA	NA	NA	57/0	57/0	2.036	1	0.154	55/2	57/0
	rs2687110	1.524	2	0.467	19/29/9	23/29/5	1.195	1	0.274	67/47	75/39	1.303	1	0.254	67/47	75/39	1.303	1	0.254	48/9	52/5	0.603	1	0.437	19/38	23/34
	rs2687116	1.694	2	0.429	3/20/34	1/25/31	0.025	1	0.875	26/88	27/87	0.028	1	0.868	26/88	27/87	0.322	1	0.570	23/34	26/31	1.036	1	0.309	3/54	1/56
	rs28988583	0.049	1	0.826	0/14/43	0/13/44	0.042	1	0.838	14/100	13/101	0.049	1	0.826	14/100	13/101	0.049	1	0.826	14/43	13/44	NA	NA	NA	0/57	0/57
	rs3735451	2.257	2	0.324	38/17/2	37/20/0	0.030	1	0.863	93/21	94/20	0.033	1	0.856	93/21	94/20	2.036	1	0.154	55/2	57/0	0.039	1	0.844	38/19	37/20
	rs746971934	1.579	1	0.209	0/7/50	0/12/45	1.435	1	0.231	7/107	12/102	1.579	1	0.209	7/107	12/102	1.579	1	0.209	7/50	12/45	NA	NA	NA	0/57	0/57
	rs778270963	2.598	1	0.107	0/14/43	0/22/35	2.111	1	0.146	14/100	22/92	2.598	1	0.107	14/100	22/92	2.598	1	0.107	14/43	22/35	NA	NA	NA	0/57	0/57
	rs915268104	0.691	1	0.406	0/9/48	0/6/51	0.642	1	0.423	9/105	6/108	0.691	1	0.406	9/105	6/108	0.691	1	0.406	9/48	6/51	NA	NA	NA	0/57	0/57
	CYP3A5*3	4.311	2	0.116	3/12/42	0/18/39	0.000	1	1.000	18/96	18/96	0.000	1	1.000	18/96	18/96	0.384	1	0.536	15/42	18/39	3.081	1	0.079	3/54	0/57
	CYP3A5*6	1.227	2	0.541	2/16/39	2/11/44	0.844	1	0.358	20/94	15/99	0.775	1	0.379	20/94	15/99	1.108	1	0.293	18/39	13/44	0.000	1	1.000	2/55	2/55
CYP3A5	NOVEL_99666556	1.303	1	0.254	0/5/52	0/9/48	1.218	1	0.270	5/109	9/105	1.303	1	0.254	5/109	9/105	1.303	1	0.254	5/52	9/48	NA	NA	NA	0/57	0/57
	NOVEL_99672211	1.883	1	0.170	53/4/0	56/1/0	1.840	1	0.175	110/4	113/1	1.883	1	0.170	110/4	113/1	NA	NA	NA	57/0	57/0	1.883	1	0.170	53/4	56/1

NOVEL_99672251	3.353	1	0.067	0/3/54	0/9/48	3.167	1	0.075	3/111	9/105	3.353	1	0.067	3/111	9/105	3.353	1	0.067	3/54	9/48	NA	NA	NA	0/57	0/57
NOVEL_99672254	0.162	1	0.687	0/19/38	0/17/40	0.132	1	0.716	19/95	17/97	0.162	1	0.687	19/95	17/97	0.162	1	0.687	19/38	17/40	NA	NA	NA	0/57	0/57
NOVEL_99672285	0.371	1	0.542	0/19/38	0/16/41	0.304	1	0.582	19/95	16/98	0.371	1	0.542	19/95	16/98	0.371	1	0.542	19/38	16/41	NA	NA	NA	0/57	0/57
rs1039108105	4.728	1	0.030	0/6/51	0/15/42	4.248	1	0.039	6/108	15/99	4.728	1	0.030	6/108	15/99	4.728	1	0.030	6/51	15/42	NA	NA	NA	0/57	0/57
rs1458424958	0.373	1	0.542	0/7/50	0/5/52	0.352	1	0.553	7/107	5/109	0.373	1	0.542	7/107	5/109	0.373	1	0.542	7/50	5/52	NA	NA	NA	0/57	0/57
rs1462057054	0.632	1	0.427	0/17/40	0/21/36	0.505	1	0.477	17/97	21/93	0.632	1	0.427	17/97	21/93	0.632	1	0.427	17/40	21/36	NA	NA	NA	0/57	0/57
rs2040992	NA	NA	NA	57/0/0	57/0/0	NA	NA	NA	114/0	114/0	NA	NA	NA	114/0	114/0	NA	NA	NA	57/0	57/0	NA	NA	NA	57/0	57/0
rs41303322	0.211	1	0.646	0/13/44	0/11/46	0.186	1	0.666	13/101	11/103	0.211	1	0.646	13/101	11/103	0.211	1	0.646	13/44	11/46	NA	NA	NA	0/57	0/57
rs4646453	1.734	2	0.420	1/6/50	1/11/45	1.311	1	0.252	8/106	13/101	1.183	1	0.277	8/106	13/101	1.579	1	0.209	7/50	12/45	0.000	1	1.000	1/56	1/56
rs6976017	6.190	2	0.045	3/15/39	0/25/32	0.436	1	0.509	21/93	25/89	0.479	1	0.489	21/93	25/89	1.830	1	0.176	18/39	25/32	3.081	1	0.079	3/54	0/57
rs8175345	1.059	2	0.589	1/7/49	1/11/45	0.805	1	0.370	9/105	13/101	0.736	1	0.391	9/105	13/101	0.970	1	0.325	8/49	12/45	0.000	1	1.000	1/56	1/56

Supplementary Table 5: Tests of association between genotype and outcome of 70 detected single nucleotide polymorphisms (SNPs) with a minor allele frequency (MAF) > 5%. The significance of each SNP via the genotypic 2 d.f in χ2 test, in addition to the 1 d.f χ2 test of the heterozygous and homozygous alternate genotypes. This included the odds ratio (OR) as calculated for each outcome of a PZQ treatment.

Enzyme	SNP	Genotype	Basic Association Test			Odds Ratios					
						Not Cleared Infection			Cleared Infection		
			χ2 Value	*DF*	*P* value	OR	L95	U95	OR	L95	U95
CYP1A2	CYP1A2*1C	*aa* vs *a/A* vs *A/A*	2.209	2	0.331						
		a/A	0.343	1	0.558	0.795	0.37	1.712	1.257	0.584	2.706
		A/A	2.171	1	0.141	2.484	0.718	8.592	0.403	0.116	1.392
	CYP1A2*1F	*aa* vs *a/A* vs *A/A*	0.763	2	0.683						
		a/A	0.143	1	0.706	1.153	0.55	2.418	0.867	0.414	1.818
		A/A	0.162	1	0.687	1.176	0.534	2.594	0.85	0.385	1.874
	CYP1A2*1K	*aa* vs *a/A* vs *A/A*	2.681	2	0.262						
		a/A	2.669	1	0.102	0.457	0.176	1.184	2.188	0.844	5.667
		A/A	0	1	1	1	0.061	16.386	1	0.061	16.386
	NOVEL_74747713	*a/A*	2.931	1	0.087	2.819	0.829	9.589	0.355	0.104	1.207
	NOVEL_74747716	*a/A*	0.622	1	0.43	0.658	0.231	1.87	1.52	0.535	4.32
	NOVEL_74747757	*a/A*	1.362	1	0.243	1.736	0.683	4.414	0.576	0.227	1.464
	NOVEL_74747828	*a/A*	0.063	1	0.802	1.135	0.423	3.043	0.881	0.329	2.363
	NOVEL_74753482	*a/A*	0.046	1	0.83	1.097	0.472	2.55	0.912	0.392	2.119
	NOVEL_74753485	*aa* vs *a/A* vs *A/A*	2.895	1	0.089						
		a/A	2.895	1	0.089	0.478	0.203	1.128	2.091	0.887	4.931
		A/A	2.895	1	0.089	2.091	0.887	4.931	0.478	0.203	1.128
	NOVEL_74753512	*a/A*	0	1	1	1	0.434	2.302	1	0.434	2.302
	NOVEL_74753515	*a/A*	0.291	1	0.59	1.339	0.462	3.882	0.747	0.258	2.164
	rs1022705765	*a/A*	1.9	1	0.168	1.905	0.756	4.8	0.525	0.208	1.323
	rs1450415112	*a/A*	2.028	1	0.154	0.501	0.192	1.31	1.994	0.763	5.21
	rs951840747	*a/A*	6.591	1	0.01	0.277	0.1	0.767	3.613	1.304	10.004
CYP2C9	CYP2C9*9	*aa* vs *a/A* vs *A/A*	0	2	1						
		a/A	0	1	1	1	0.426	2.346	1	0.426	2.346

		A/A	0	1	1	1	0.061	16.386	1	0.061	16.386
	NOVEL_94956743	*a/A*	0.069	1	0.793	1.148	0.409	3.224	0.871	0.31	2.444
	NOVEL_94977838	*a/A*	0.326	1	0.568	0.721	0.233	2.228	1.388	0.449	4.291
		aa vs *a/A* vs *A/A*	0.42	2	0.811						
	rs2017319	*a/A*	0.051	1	0.821	1.108	0.456	2.692	0.903	0.371	2.193
		A/A	0.342	1	0.558	2.036	0.179	23.11	0.491	0.043	5.573
		aa vs *a/A* vs *A/A*	0.674	2	0.714						
	rs75541073	*a/A*	0.399	1	0.528	0.766	0.335	1.753	1.305	0.57	2.988
		A/A	0.342	1	0.558	2.036	0.179	23.11	0.491	0.043	5.573
		aa vs *a/A* vs *A/A*	0.419	2	0.811						
	rs9332127	*a/A*	0.416	1	0.519	0.757	0.325	1.765	1.321	0.567	3.08
		A/A	0	1	1	1	0.061	16.386	1	0.061	16.386
		aa vs *a/A* vs *A/A*	0.42	2	0.811						
	rs9332232	*a/A*	0.051	1	0.821	1.108	0.456	2.692	0.903	0.371	2.193
		A/A	0.342	1	0.558	2.036	0.179	23.11	0.491	0.043	5.573
		aa vs *a/A* vs *A/A*	1.493	2	0.474						
	rs9332241	*a/A*	1.49	1	0.222	2.163	0.613	7.638	0.462	0.131	1.632
		A/A	0	1	1	1	0.061	16.386	1	0.061	16.386
		aa vs *a/A* vs *A/A*	1.929	2	0.381						
	CYP2C19*17	*a/A*	1.9	1	0.168	0.525	0.208	1.323	1.905	0.756	4.8
		A/A	0	1	1	1	0.193	5.177	1	0.193	5.177
	CYP2C19*27	*a/A*	0.044	1	0.833	1.093	0.479	2.494	0.915	0.401	2.089
	NOVEL_94779010	*a/A*	0.691	1	0.406	0.627	0.208	1.896	1.594	0.528	4.815
		aa vs *a/A* vs *A/A*	1.591	2	0.451						
CYP2C19	rs17879992	*a/A*	1.579	1	0.209	1.905	0.69	5.258	0.525	0.19	1.449
		A/A	0	1	1	1	0.136	7.354	1	0.136	7.354
		aa vs *a/A* vs *A/A*	1.053	2	0.591						
	rs17884938	*a/A*	0.063	1	0.802	0.881	0.329	2.363	1.135	0.423	3.043
		A/A	1.009	1	0.315						
	rs17885567	*a/A*	0.087	1	0.768	0.84	0.264	2.676	1.19	0.374	3.789
	rs4244285	*aa* vs *a/A* vs *A/A*	1.047	2	0.592						

	a/A	0.046	1	0.83	1.097	0.472	2.55	0.912	0.392	2.119
	A/A	1.036	1	0.309	0.321	0.032	3.186	3.111	0.314	30.84
	aa vs *a/A* vs *A/A*	0.429	2	0.807						
rs4917623	*a/A*	0.063	1	0.802	0.881	0.329	2.363	1.135	0.423	3.043
	A/A	0.342	1	0.558	0.491	0.043	5.573	2.036	0.179	23.11
	aa vs *a/A* vs *A/A*	0.182	2	0.913						
rs4986894	*a/A*	0.181	1	0.671	0.834	0.362	1.923	1.199	0.52	2.763
	A/A	0	1	1	1	0.061	16.386	1	0.061	16.386
rs76267522	*a/A*	0.373	1	0.542	1.456	0.434	4.89	0.687	0.204	2.307
	aa vs *a/A* vs *A/A*	3.26	2	0.196						
CYP2D6*17	*a/A*	0.199	1	0.655	1.221	0.508	2.935	0.819	0.341	1.969
	A/A	2.815	1	0.093	5.385	0.609	47.632	0.186	0.021	1.643
rs17002853	*a/A*	0.781	1	0.377	0.589	0.18	1.923	1.698	0.52	5.545
	aa vs *a/A* vs *A/A*	6.105	2	0.047						
rs28371702	*a/A*	6.049	1	0.014	0.387	0.18	0.831	2.583	1.203	5.546
	A/A	5.092	1	0.024	2.368	1.113	5.037	0.422	0.199	0.898
NOVEL_99761942	*a/A*	2.375	1	0.123	0.441	0.153	1.272	2.267	0.786	6.535
NOVEL_99762108	*a/A*	3.241	1	0.072	0.481	0.216	1.074	2.078	0.931	4.635
NOVEL_99762115	*a/A*	1.728	1	0.189	0.492	0.168	1.437	2.033	0.696	5.935
NOVEL_99762116	*a/A*	1.728	1	0.189	0.492	0.168	1.437	2.033	0.696	5.935
NOVEL_99764034	*a/A*	1.961	1	0.161	0.568	0.257	1.258	1.76	0.795	3.897
NOVEL_99764099	*a/A*	0.063	1	0.802	1.135	0.423	3.043	0.881	0.329	2.363
NOVEL_99764101	*a/A*	0.264	1	0.607	1.303	0.474	3.586	0.767	0.279	2.111
NOVEL_99766252	*a/A*	0.373	1	0.542	1.456	0.434	4.89	0.687	0.204	2.307
NOVEL_99784449	*a/A*	0.041	1	0.839	1.086	0.49	2.408	0.921	0.415	2.042
NOVEL_99784471	*a/A*	0.199	1	0.655	0.819	0.341	1.969	1.221	0.508	2.935
rs1006181087	*a/A*	1.579	1	0.209	0.525	0.19	1.449	1.905	0.69	5.258
rs12721622	*a/A*	0.225	1	0.635	0.798	0.314	2.029	1.253	0.493	3.188
	aa vs *a/A* vs *A/A*	2.036	1	0.154						
rs1479820461	*a/A*	2.036	1	0.154						
	A/A	2.036	1	0.154						

		aa vs *a/A* vs *A/A*	1.524	2	0.467						
	rs2687110	*a/A*	0	1	1	1	0.48	2.084	1	0.48	2.084
		A/A	1.303	1	0.254	1.95	0.61	6.23	0.513	0.161	1.638
		aa vs *a/A* vs *A/A*	1.694	2	0.429						
	rs2687116	*a/A*	0.918	1	0.338	0.692	0.325	1.472	1.445	0.68	3.074
		A/A	1.036	1	0.309	3.111	0.314	30.84	0.321	0.032	3.186
	rs28988583	*a/A*	0.049	1	0.826	1.102	0.464	2.615	0.907	0.382	2.153
		aa vs *a/A* vs *A/A*	2.257	2	0.324						
	rs3735451	*a/A*	0.36	1	0.548	0.786	0.358	1.726	1.272	0.58	2.791
		A/A	0.039	1	0.843	1.081	0.499	2.344	0.925	0.427	2.006
	rs746971934	*a/A*	1.579	1	0.209	0.525	0.19	1.449	1.905	0.69	5.258
	rs778270963	*a/A*	2.598	1	0.107	0.518	0.232	1.159	1.931	0.863	4.318
	rs915268104	*a/A*	0.691	1	0.406	1.594	0.528	4.815	0.627	0.208	1.896
		aa vs *a/A* vs *A/A*	4.311	2	0.116						
	CYP3A5*3	*a/A*	1.629	1	0.202	0.578	0.248	1.348	1.731	0.742	4.037
		A/A	3.081	1	0.079						
		aa vs *a/A* vs *A/A*	1.227	2	0.541						
	CYP3A5*6	*a/A*	1.213	1	0.271	1.632	0.68	3.917	0.613	0.255	1.471
		A/A	0	1	1	1	0.136	7.354	1	0.136	7.354
	NOVEL_99666556	*a/A*	1.303	1	0.254	0.513	0.161	1.638	1.95	0.61	6.23
		aa vs *a/A* vs *A/A*	1.883	1	0.17						
CYP3A5	NOVEL_99672211	*a/A*	1.883	1	0.17	4.226	0.458	39.041	0.237	0.026	2.186
		A/A	1.883	1	0.17	0.237	0.026	2.186	4.226	0.458	39.041
	NOVEL_99672251	*a/A*	3.353	1	0.067	0.296	0.076	1.158	3.375	0.863	13.193
	NOVEL_99672254	*a/A*	0.162	1	0.687	1.176	0.534	2.594	0.85	0.385	1.874
	NOVEL_99672285	*a/A*	0.371	1	0.542	1.281	0.577	2.846	0.78	0.351	1.734
	rs1039108105	*a/A*	4.728	1	0.03	0.329	0.117	0.924	3.036	1.083	8.512
	rs1458424958	*a/A*	0.373	1	0.542	1.456	0.434	4.89	0.687	0.204	2.307
	rs1462057054	*a/A*	0.632	1	0.427	0.729	0.333	1.593	1.373	0.628	3
	rs2040992	*A/A*									
	rs41303322	*a/A*	0.211	1	0.646	1.236	0.501	3.048	0.809	0.328	1.997

rs4646453	*aa* vs *a/A* vs *A/A*	1.734	2	0.42						
	a/A	1.728	1	0.189	0.492	0.168	1.437	2.033	0.696	5.935
	A/A	0	1	1	1	0.061	16.386	1	0.061	16.386
rs6976017	*aa* vs *a/A* vs *A/A*	6.19	2	0.045						
	a/A	3.851	1	0.05	0.457	0.208	1.005	2.188	0.995	4.811
	A/A	3.081	1	0.079						
rs8175345	*aa* vs *a/A* vs *A/A*	1.059	2	0.589						
	a/A	1.056	1	0.304	0.585	0.209	1.638	1.708	0.611	4.778
	A/A	0	1	1	1	0.061	16.386	1	0.061	16.386

Supplementary Table 6: The single nucleotide polymorphisms (SNPs) found to be significant associated with an individual clearing infection via basic association analysis. Association tests were used to determine the genotype significantly associated with PZQ efficacy using PLINK; these are listed below each genetic model. The frequency of the allele/genotype for each model is listed, with the frequency separated by treatment outcome.

Enzyme	SNP	MAF (%)		Genotypic Association						Allelic Association				Cochran-Armitage Trend				Dominant Model				Recessive Model			
				Fishers Exact test		$\chi2_{Yates}$ test		*AA vs aA vs aa*		$\chi2_{Yates}$ test		*A vs a*		T^2 test		*A vs a*		$\chi2_{Yates}$ test		*aA+AA vs aa*		$\chi2_{Yates}$ test		*AA vs aa+aA*	
		NC	*C*	*DF*	*P*	Genotype	P_{adj}	*NC*	*C*	*DF*	P_{adj}	NC	C	*DF*	*P*	NC	C	*DF*	P_{adj}	NC	C	*DF*	P_{adj}	NC	C
CYP1A2	rs951840747	2.632	7.456	1	**0.018**	***CT***<br>*TT*	**0.020**<br>NA	0/6/51	0/17/40		**0.028**	6/108	17/97		**0.010**	6/108	17/97		**0.020**	6/51	17/40		NA	0/57	0/57
CYP2D6	rs28371702	40.351	35.326	2	**0.031**	***AC***<br>***CC***	**0.023**<br>**0.039**	37/18/2	25/31/1		0.122	92/22	81/33		0.061	92/22	81/33		1.000	55/2	56/1		**0.039**	37/20	25/32
										1				1				1				1			
CYP3A5	rs1039108105	2.632	6.579	1	0.052	*GC*<br>*CC*	0.053<br>NA	0/6/51	0/15/42		0.067	6/108	15/99		**0.030**	6/108	15/99		0.53	6/51	15/42		NA	0/57	0/57
	rs6976017	9.211	10.965	2	**0.041**	*GA*<br>*AA*	0.077<br>0.240	3/15/39	0/25/32		0.621	21/93	25/89		0.489	21/93	25/89		0.246	18/39	25/32		0.242	3/54	0/57

Bold indicates the SNP genotype that were determined to be significantly associated with PZQ efficacy. The significance threshold is a $P \leq 0.05$, with the P_{adj} obtained from $\chi2_{Yates}$ test representing an adjusted P-value accounting for Type 1 continuity correction. CYP: Cytochrome P450 enzyme, NC: Not cleared group, C: Cleared group.

Supplementary Table 7: SNP pairs that were found to have evidence of strong LD in this Zimbabwean population but were not present in a haplotype block.

Chromosome	SNP 1	SNP 2	D'	CI (95%)	LOD	r^2	Evidence of LD
7	rs4646453	rs8175345	1	[0.9 ,1]	23.55	0.95	STRONG
	CYP3A5*3	rs1458424958	1	[0.73 ,1]	7.46	0.296	STRONG
	rs6976017	Novel-14	1	[0.7 ,1]	6.24	0.22	STRONG
10	rs4986894	CYP2C19*2	1	[0.92 ,1]	28.75	0.905	STRONG
	rs17884938	rs9332232	1	[0.86 ,1]	16.85	0.645	STRONG
	rs17884938	rs2017319	1	[0.86 ,1]	16.85	0.645	STRONG
	rs17879992	rs9332241	1	[0.82 ,1]	13.92	0.562	STRONG
	rs17885567	rs9332127	1	[0.76 ,1]	8.43	0.357	STRONG
	rs4986894	rs17885567	1	[0.76 ,1]	8.22	0.345	STRONG
	CYP2C19*2	rs17885567	1	[0.75 ,1]	7.93	0.312	STRONG
	rs17885567	rs75541073	1	[0.75 ,1]	7.62	0.312	STRONG
15	CYP1A2*1C	CYP1A2*1F	1	[0.89 ,1]	16.27	0.362	STRONG

D' is the value of D prime between the two loci; LOD is the log of the likelihood odds ratio, a measure of confidence; r^2 is the correlation coefficient between the two loci; CI (95%) is 95% confidence interval bounds on D'.

Supplementary Table 8: Linkage disequilibrium (LD) statistics for the SNPs in the three haplotype blocks across chromosomes 7 and 10. D' is the value of D prime between the two loci; LOD is the log of the likelihood odds ratio, a measure of confidence; r^2 is the correlation coefficient between the two loci; CI (95%) is 95% confidence interval bounds on D'. All pairwise comparisons of SNPs were < 500 kb apart, and all SNPs that violated the HWE were removed from LD analysis.

Chromosome	Gene	SNP 1	SNP 2	D'	CI (95%)	LOD	r^2	Evidence of LD
7	CYP3A4	Novel-18	rs1006181087	1	[0.87 ,1]	18.37	0.886	STRONG
		Novel-19	rs1006181087	1	[0.87 ,1]	18.37	0.886	STRONG
		rs778270963	rs1006181087	1	[0.83 ,1]	11.87	0.485	STRONG
		rs778270963	rs746971934	1	[0.83 ,1]	11.87	0.485	STRONG
		Novel-17	rs1006181087	1	[0.82 ,1]	11.55	0.469	STRONG
		Novel-17	rs746971934	1	[0.82 ,1]	11.55	0.469	STRONG
		rs778270963	Novel-18	1	[0.8 ,1]	10.34	0.43	STRONG
		rs778270963	Novel-19	1	[0.8 ,1]	10.34	0.43	STRONG
		Novel-17	Novel-18	1	[0.8 ,1]	10.07	0.416	STRONG
		Novel-17	Novel-19	1	[0.8 ,1]	10.07	0.416	STRONG
10	CYP2C9	rs9332232	rs2017319	1	[0.94 ,1]	31.84	1	**COMPLETE**
		rs9332127	rs75541073	1	[0.91 ,1]	27.01	0.874	STRONG

Supplementary Table 9: Analysis of the SNPs identified in the haplotype blocks during linkage disequilibrium (LD) analysis on chromosome 7 and chromosome 10. The frequency of each haplotype in those who cleared infection and those who did not clear infection.

Chromosome	Gene	Block	SNP	Haplotype	Frequency of Cleared vs Not Cleared	χ2	P Value
7	CYP3A4	Block 1	rs778270963	CTCACC	0.868, 0.789	2.506	0.1134
			rs746971934	ACTGTA	0.053, 0.079	0.642	0.4229
			rs1006181087	ACCACC	0.053, 0.070	0.304	0.5816
			Novel-17				
			Novel-18	CCCACC	0.009, 0.018	0.337	0.5614
			Novel-19				
10	CYP2C9	Block 1	rs9332127	GG	0.842, 0.833	0.032	0.8575
			rs75541073	CA	0.132, 0.158	0.319	0.5723
				GA	0.026, 0.009	1.018	0.313
		Block 2	rs9332232	TC	0.851, 0.877	0.336	0.5621
			rs2017319	CT	0.149, 0.123	0.336	0.5621

Statistics indicate the χ2 value obtained from Pearson's χ2 analysis, and the associated *P*-value towards treatment outcome. The haplotype blocks were defined based on the Gabriel method (upper 95% CI of the D' value is ≥ 0.98, and the lower 95% CI is ≥ 0.7; minimum allele frequency, 5%) [1].

Supplementary Table 10: Predictive error rate (OOB-ER) of the random forest (RF) after backwards-purging of the dataset. Low overall OOB-ER, but relatively high OOB-ER for the individuals who had not cleared infection who had been classed as clearing infection. Here, the overall OOB-ER is relatively high (0.29) with the OOB-ER for the individuals who had not cleared infection is slightly higher (0.31).

Type of random forest: classification Number of trees: 1000<br>No. of variables tried at each split: 4<br>OOB estimate of error rate: 29.82%	**Cleared Infection (RF)**	**Not Cleared Infection (RF)**	**Class Error**
**Cleared Infection**	41 (TRUE)	16 (FALSE)	0. 2807018
**Not Cleared Infection**	18 (FALSE)	39(TRUE)	0. 3157895

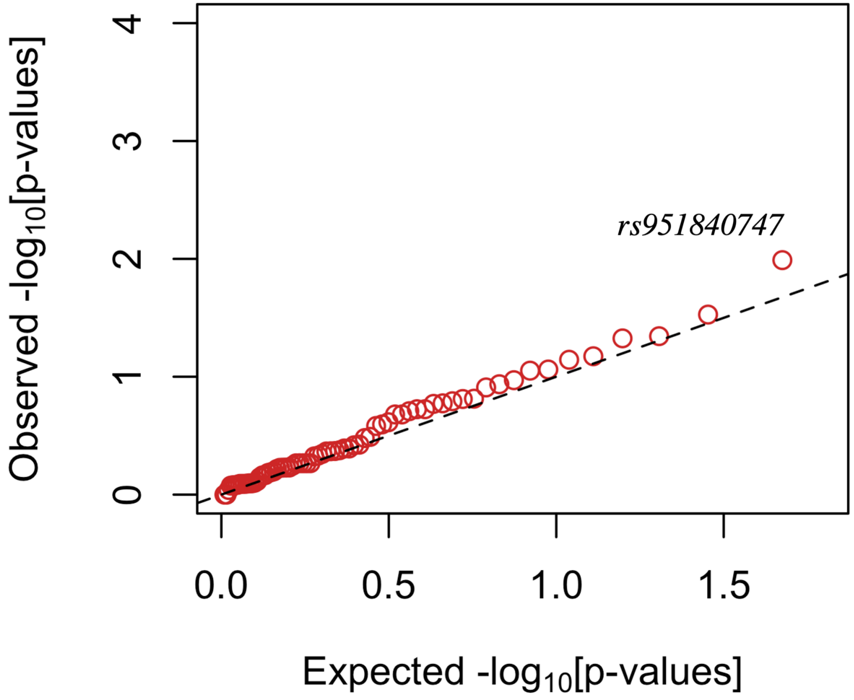

Supplementary Figure 1: Quantile plot of observed -log$_{10}$[p-values] from the genotypic association against expected -log$_{10}$[p-values] for single nucleotide polymorphisms (SNPs) detected in this study. Under the null hypothesis of no association between SNP and treatment outcome (whether an individual clears or does not clear infection) the *P*-values should follow a uniform distribution (black line, y=x). The deviation of some SNPs from the dotted line indicated stronger associations with PZQ efficacy than by chance, with rs951840747 showing the largest deviation.

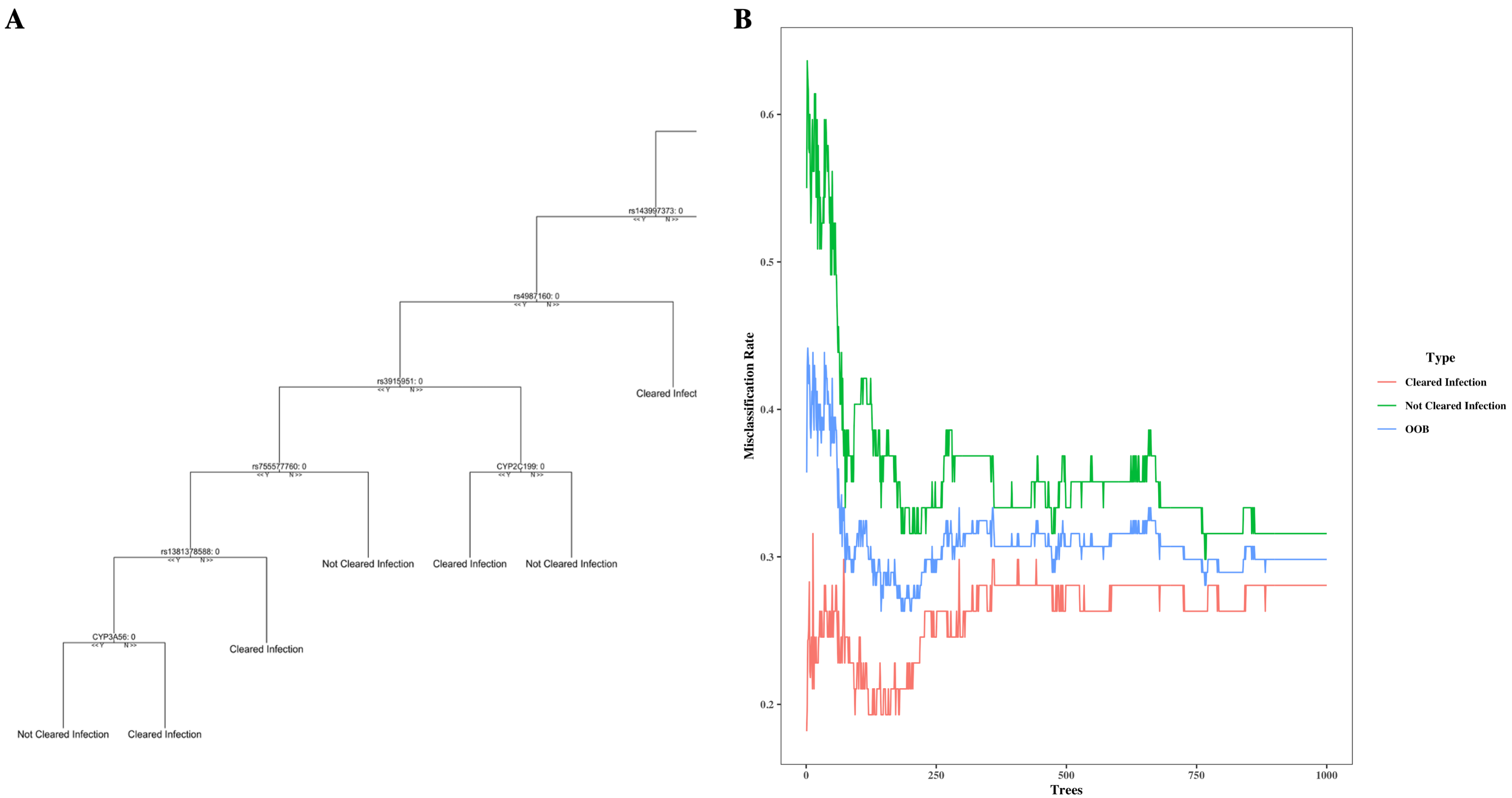

Supplementary Figure 2: The random forest (RF) built by a series of classification trees by randomly sampling a subset of the subjects to form an out-of-bag [OOB] data set, from which RF randomly selects and searches for the best SNP predictive of a PZQ treatment outcome. The optimal SNP becomes the first node in the tree and RF continues to randomly select a subset of SNPs at each node and then partition the data until a full tree is grown. **[A] Diagram illustrating the last few nodes of a classification-based RF tree to identify single nucleotide polymorphisms (SNPs) associated with a schistosomiasis treatment outcome.** Incudes examples of the classification per SNP and the respective genotype. This method reduces the variance and bias within and between the trees, improving the RF's predictive power. Genotypic information was used to assess the power of each SNP to predict whether an individual clears or not clears infection. The samples not included in the OOB data were classified using the tree, and the misclassification of those individuals provides an estimate of

the predictive error rate of the RF. **[B] The out-of-bag error per number of trees (OOB-ER).** Numerous trees were run until the OOB-ER was normalised to build the forest until the OOB-ER reaches a plateau. The importance of each SNP was then calculated based on OOB data, testing the prediction accuracy from the original OOB sample in relation to another OOB sample where the genotype of each SNP was permuted. The importance value of each SNP was then averaged across trees and analysed to ultimately determine those that best explain variation in PZQ treatment outcome.

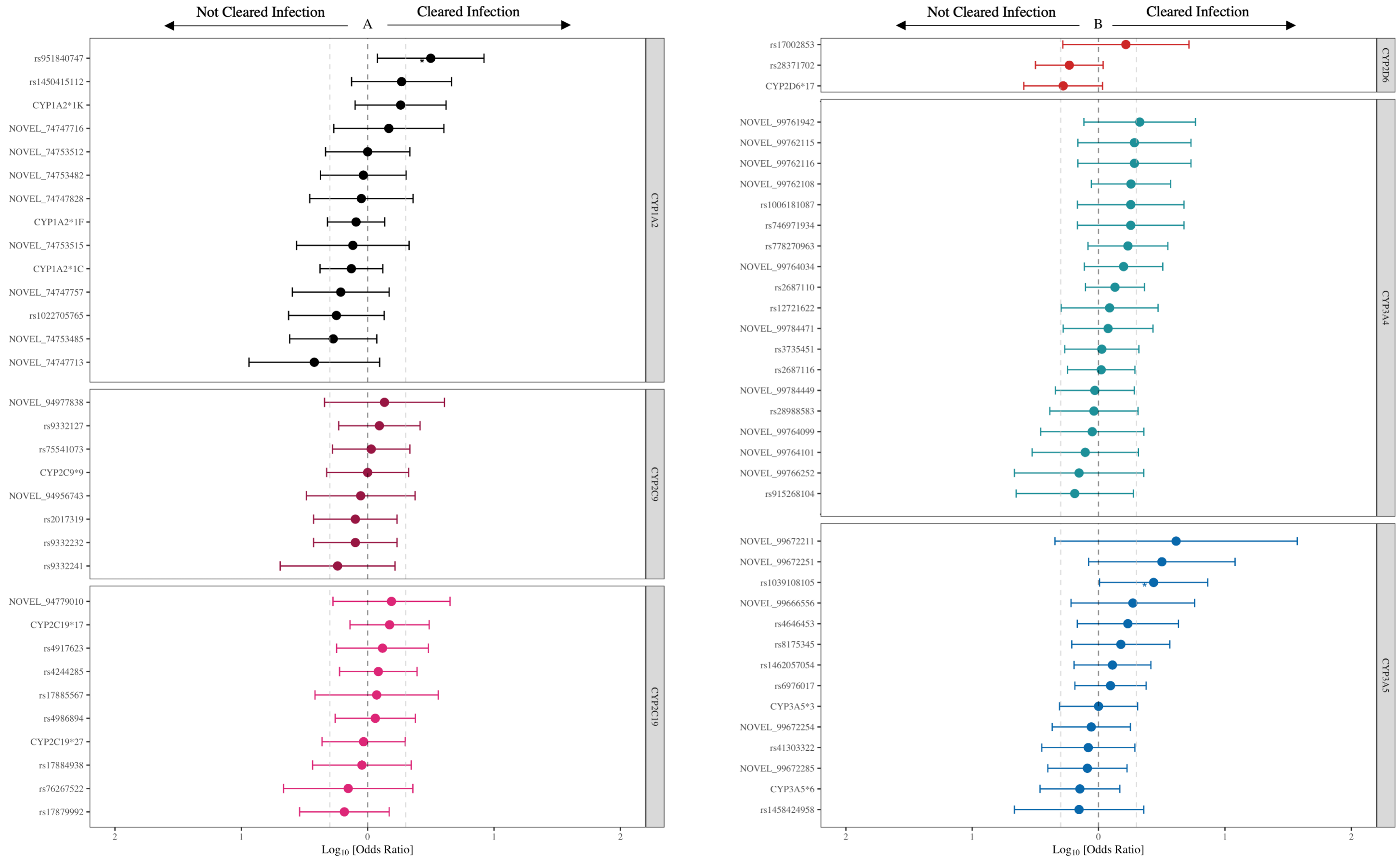

Supplementary Figure 3: Allelic Log$_{10}$(Odds Ratio) of SNPs in relation to PZQ efficacy, with each SNP indicating an increased odd of clearing (right) or not clearing (left) schistosomiasis infection. The significant Log$_{10}$(Odds Ratio)'s are labelled with (*). The use of Log$_{10}$(Odds Ratio) was used to normalise the scale of results, for every 1-unit increase, the risk is multiplied by 10. The error bars illustrate the 95% confidence interval. Panel **(A)** and **(B)** contain the SNPs from the CYP1A2/CYP2C9/CYP2C19 and CYP2D6/CYP3A4/CYP3A5 respectively. Each SNP illustrates whether the odds ratio for the alternate allele.

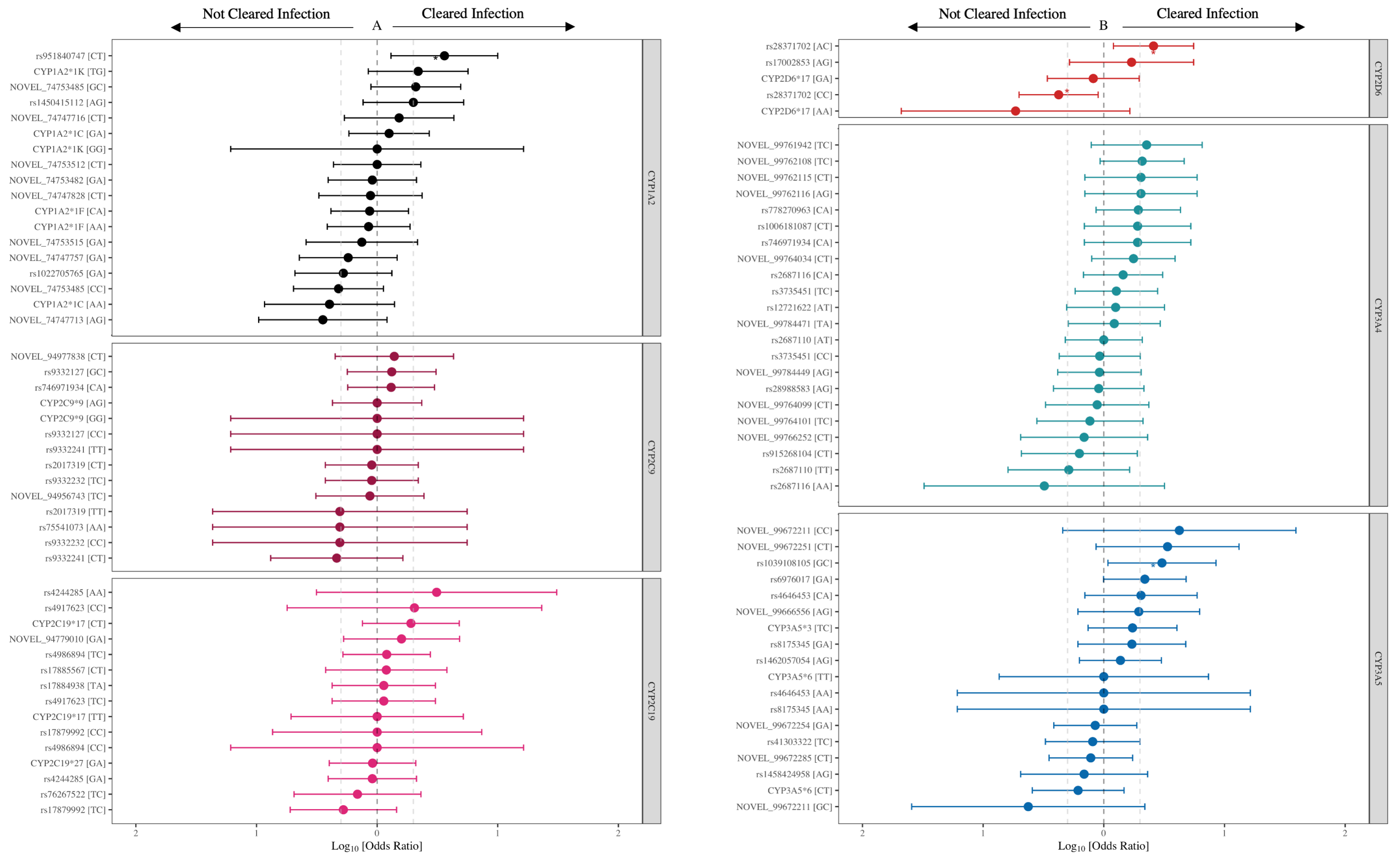

Supplementary Figure 4: Genotypic Log$_{10}$(Odds Ratio) of SNPs in relation to PZQ efficacy, with each SNP indicating an increased odd of clearing (right) or not clearing (left) schistosomiasis infection. The significant Log$_{10}$(Odds Ratio)'s are labelled with (*). The use of Log$_{10}$(Odds Ratio) was used to normalise the scale of results, for every 1-unit increase, the risk is multiplied by 10. The error bars illustrate the 95% confidence interval. Panel **(A)** and **(B)** contain the SNPs from the CYP1A2/CYP2C9/CYP2C19 and CYP2D6/CYP3A4/CYP3A5 respectively. The genotype of each SNP is displayed in parentheses, illustrating whether the odds ratio represents the heterozygous or homozygous genotype for the alternate allele.

A

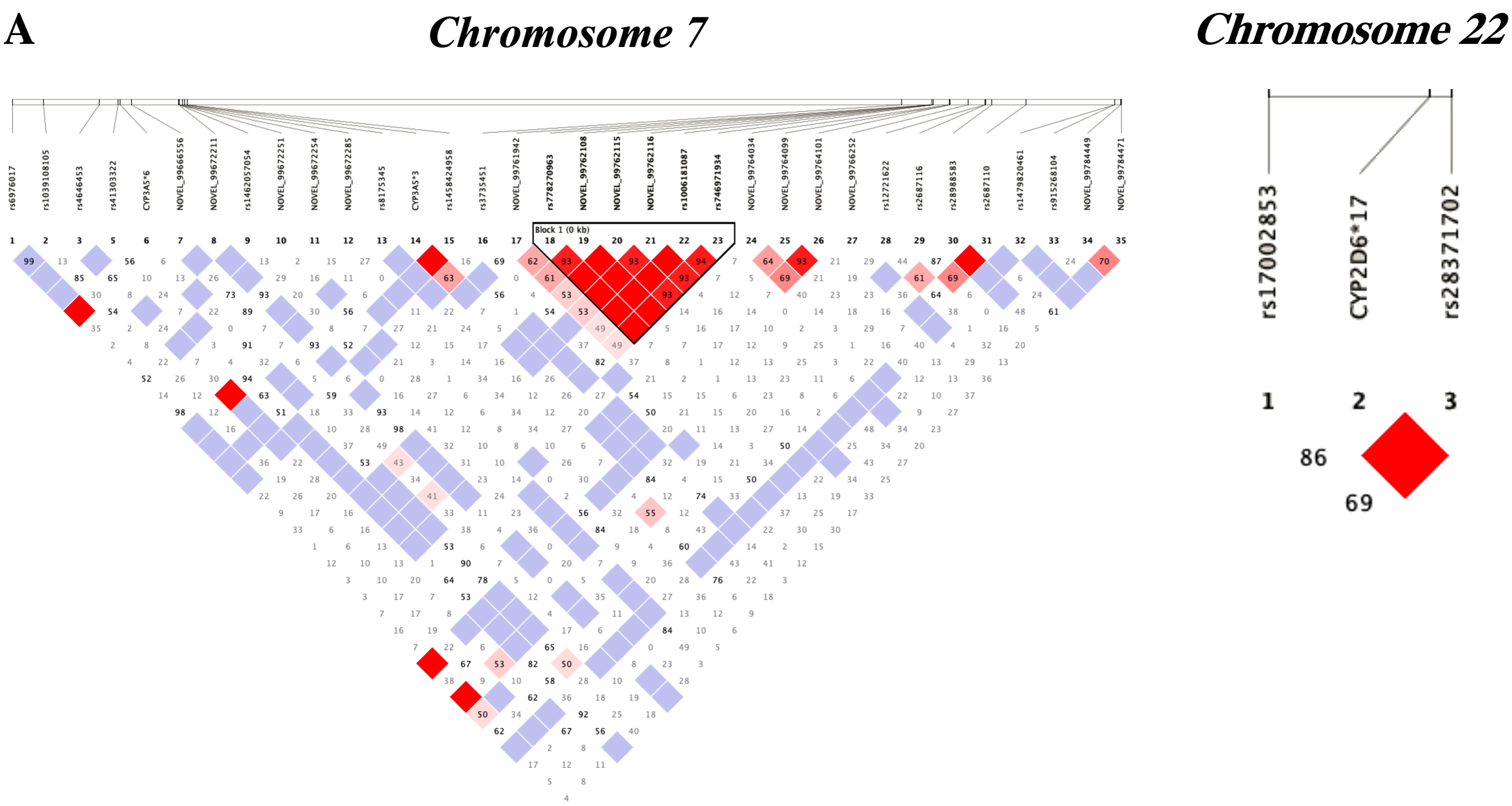

Supplementary Figure 5: (A) Linkage disequilibrium (LD) analysis of the CYP3A4 and CYP3A5 genes of chromosome 7, and CYP2D6 gene of chromosome 22. Strong LD is displayed by bright red (very strong: LOD > 2, D' = 1), moderate LD in pink/red (moderately strong: LOD > 2, D' < 1), intermediate LD is displayed by blue (LOD < 2, D' = 1), and absence of LD is displayed by white (LOD < 2, D' < 1). All pairwise comparisons of SNPs were < 500 kb apart, and all SNPs that violated the HWE were removed from LD analysis.

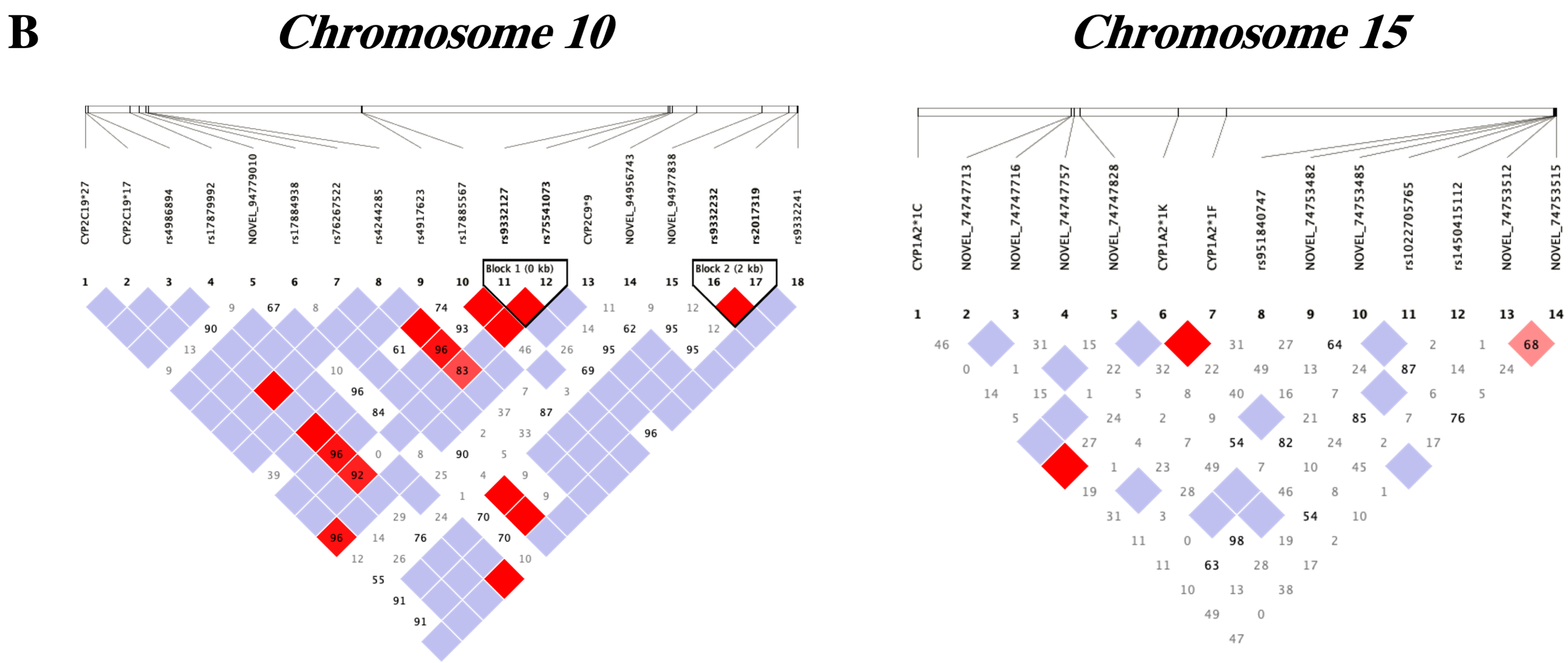

Supplementary Figure 5: (B) Linkage disequilibrium (LD) analysis of the CYP2C9 and CYP2C19 genes of chromosome 10, and CYP1A2 gene of chromosome 15. Strong LD is displayed by bright red (very strong: LOD > 2, D' = 1), moderate LD in pink/red (moderately strong: LOD > 2, D' < 1), intermediate LD is displayed by blue (LOD < 2, D' = 1), and absence of LD is displayed by white (LOD < 2, D' < 1). All pairwise comparisons of SNPs were < 500 kb apart, and all SNPs that violated the HWE were removed from LD analysis.

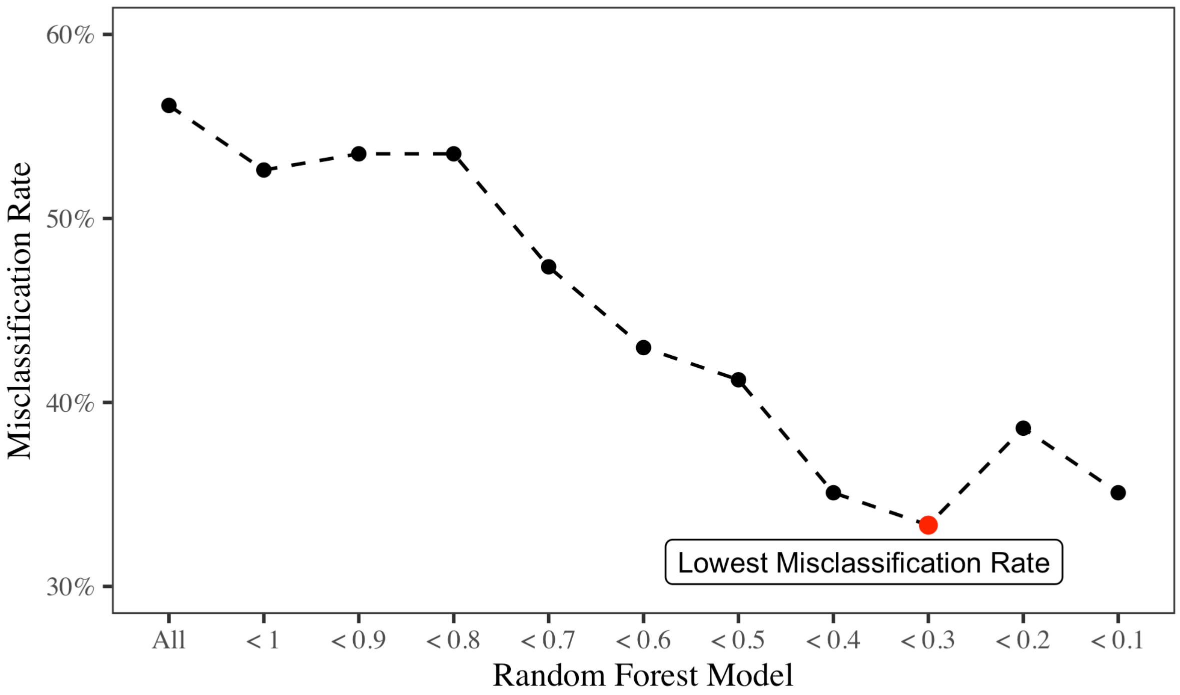

Supplementary Figure 6: A summary of each random forest (RF) model and the resulting misclassification rate (OOB-ER). The lowest misclassification rate (red dot) was observed with the *P*-value < 0.3, and therefore this was the predictive data set selected. Based on this data set, backwards-purging was continued until the lowest error rate was achieved. The RF model with the lowest error rate was selected for further discussion, which included SNPs with a $P <$ 0.3. As a RF model was built for multiple data sets, the model only including SNPs with a genotypic $P < 0.1$ was expected to be the best predictor as it contained those SNPs with the strongest association. However, this model had a higher misclassification rate of 38%, and vastly lower permutation importance values, indicating that the larger number SNPs were better predictors than just the SNPs with the strongest association.

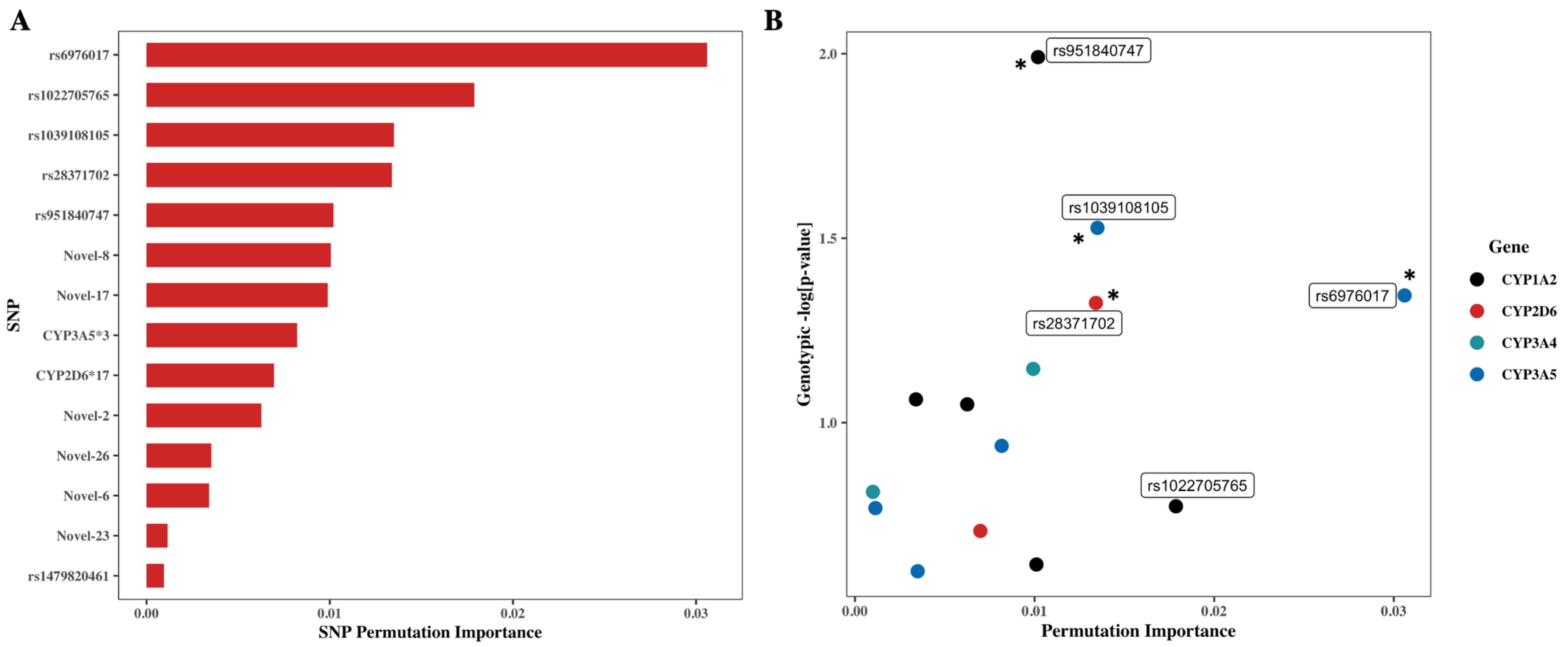

Supplementary Figure 7: (A) The most important SNPs to predict the PZQ efficacy of an individual as produced by the random forest (RF) model. This was produced using SNPs whose genotype was associated with PZQ efficacy (P < 0.3) to create a model with the lowest misclassification rate**. (B) Genotypic association and predictive permutation importance of SNPs in the RF model (P <0.3).** The permutation importance is produced by the "MeanAccuracyDecrease" values of the RF model, the genotypic association was produced by a -$\log_{10}$[P-value] produced by a $\chi 2$ test of SNP genotype (*aa vs aA vs AA*) against treatment outcome (clearing infection or not clearing infection). Those SNPs labelled were those who were found to be designated in the top 5 most important for predicting PZQ efficacy, with those who were also significant via the univariate analysis labelled by *.
